# A donor PD-1^+^CD8^+^ T_SCM_-like regulatory subset mobilized by G-CSF alleviates recipient acute graft-versus-host-disease

**DOI:** 10.1038/s41392-025-02183-1

**Published:** 2025-04-02

**Authors:** Dan Liu, Xue Wang, Yuheng Han, Jing Wang, Yidan Sun, Yafei Hou, Qian Wu, Cong Zeng, Xuping Ding, Yingjun Chang, Jiong Hu, Xiaojun Huang, Liming Lu

**Affiliations:** 1 Shanghai Institute of Immunology, Shanghai Jiao Tong University School of Medicine, Shanghai, China; 2 Peking University People’s Hospital, Peking University Institute of Hematology, Beijing Key Laboratory of Hematopoietic Stem Cell Transplantation, National Clinical Research Center for Hematologic Disease, Beijing, China; 3 Blood and Marrow Transplantation Center, Department of Hematology, Shanghai Institute of Hematology, Ruijin Hospital, Shanghai Jiao Tong University School of Medicine, Shanghai, China

**Keywords:** Bone marrow transplantation, Transplant immunology

## Abstract

Donor selection determines the occurrence of acute graft-versus-host-disease (aGVHD) following allogeneic hematopoietic stem cell transplantation (allo-HSCT). To optimize the current clinical donor selection criteria and identify putative donor lymphocyte subsets associated with better recipient outcomes, we analyzed the peripheral CD4^+^ and CD8^+^ subsets in 80 granulocyte colony-stimulating factor (G-CSF) mobilized donors and examined the aGVHD incidence of the corresponding 80 haploidentical and identical allo-HSCT recipients. The G-CSF-induced expansion of subsets varied among donors. We discovered a novel PD-1^+^CD8^+^CD45RA^+^CCR7^+^ T lymphocyte subset in suitable donors that was significantly correlated with lower incidence of aGVHD and post-transplant anti-infection. The anti-aGVHD activity of this subset was confirmed in a validation cohort (*n* = 30). Single-cell RNA sequencing revealed that this T cell subset exhibited transcriptomic features of stem cell-like memory T cell (T_SCM_) with both Treg and Teff activities which indicated its dual functions in aGVHD inhibition and graft-versus-leukemia (GVL) effect. Intriguingly, upon G-CSF mobilization, the donor PD-1^+^CD8^+^ T_SCM_-like regulatory cells increased the PD-1 expression in a BCL6-dependent manner. Next, we showed that the mouse counterpart of this subset (PD-1^+^CD8^+^CD44^−^CD62L^+^) ameliorated aGVHD, and confirmed the existence of this subset in clinical recipients. In summary, we, for the first time, identified a novel donor peripheral T cell subset suppressing aGVHD while promoting the immune reconstitution of recipients. It may serve as an indicator for optimal haploidentical and identical donor selection. Importantly, the dual Treg and Teff function of these T cells makes it a promising treatment for not only aGVHD but also auto-immune diseases.

## Introduction

Allogeneic hematopoietic stem cell transplantation (allo-HSCT) is the most effective treatment for malignant hematological diseases.^1–3^ The process begins with G-CSF treatment of donors which mobilizes the bone marrow stem cells into the peripheral blood.^4–8^ However, acute graft-versus-host-disease (aGVHD) remains a major life-threatening complication after allo-HSCT even when human leukocyte antigens (HLA) genotype matching is identical or haploidentical. 30–50% of patients will develop aGVHD after transplantation which occurs when donor alloreactive T cells in grafts recognize recipient’s tissues as foreign and subsequently attack host tissues.^9–11^ Removal of T cells from donor grafts reduces aGVHD but increases the risk of graft failure, opportunistic infection and relapse due to decreased graft-versus-leukemia (GVL) effect.^12–14^ On the other hand, T cells play essential roles in allo-HSCT recipient immune reconstitution.^15^ Immunosuppressive drugs are commonly used to treat GVHD but often cause drug-related toxicities, severe infection and mortality. Therefore, selecting suitable donors whose T cells can alleviate GVHD meanwhile promoting the GVL and anti-infectious activities is critical for optimal outcomes of recipients after transplantation.

At present, allo-HSCT donors are primarily selected based on HLA matching.^16^ However, to increase the potential donor pool, haploidentical donors (parents, siblings and children) are now widely used and become the major selection criteria.^17^ Haplo-HSCT takes up a proportion of 63% of all allo-HSCT cases in China.^18^ It is worth noting that some clinically used selection criteria, for example, younger male donors, are based mainly on correlation studies and can cause heterogeneous patient outcomes due to the lack of mechanistic studies.^19,20^ Therefore, it is necessary to develop more accurate and comprehensive criteria for selecting suitable donors. Besides the effort to identify prognostic aGVHD biomarkers in recipients,^21,22^ it is more relevant to discover donor markers or aGVHD-inhibitory specific immune cell subsets for improved transplantation. It was reported that higher CD8^+^ T cell population in the graft predicted better survival for allo-HSCT recipients^23^ and that increased donor NK cells correlated with lower aGVHD.^24^ Nonetheless, these potential donor biomarkers have not been validated or approved for clinical applications.

Granulocyte colony-stimulating factor (G-CSF) is a classic cytokine^4^ and is used clinically to increase peripheral white blood cells (WBC) in chemoradiotherapy patients or to mobilize donor CD34^+^ hematopoietic stem cells (HSC) and other immune cells in allo-HSCT transplantations.^5–8^ The allo-HSCT G-CSF-primed peripheral blood stem cell (G-PBSC) graft is a mixture of mobilized cells which usually contain ~7.8 × 10^6^/kg CD34^+^ cells and ~8 × 10^8^/kg CD45^+^ (~100 folds more than the CD34^+^ cells) immune cells.^25^ G-CSF mobilization in healthy donors has been shown to cause immune responses including promoting myeloid-biased differentiation of lymphoid-primed multipotent progenitors in the bone marrow,^26^ negatively modulating innate immune cells,^27–29^ directly restraining activation of T cells,^30^ inducing Treg differentiation^31,32^ and balancing Th1/Th2 subsets.^33–35^ Indeed, donor G-CSF-primed bone marrow (G-BM) and G-PBSC are known to correlate with aGVHD alleviation and immune tolerance in recipients.^36–39^ However, it is not clear which particular donor immune cell subset is causally involved in reducing the consequences of aGVHD. Identification of donor delicate subset mobilized by G-CSF would facilitate favorable prognosis in allo-HSCT recipients. We previously reported using single-cell RNA sequencing (scRNA-seq) analysis in G-BM that G-CSF directly induced the expression of immunosuppressive genes in donor T cells and suppressed T cell activation.^26,30^ Nevertheless, no particular donor G-BM or G-PBSC lymphocyte subset causally involved in the balance of the aGVHD and the GVL or the inhibition of post-transplant infection has been identified. Therefore, finding and characterizing donor delicate subsets, including their surface markers, chemokine receptors, driven transcription factors and functional molecules, will greatly benefit the selection of suitable donors and the development for effective cell-based therapy for aGVHD. Programmed cell death protein-1 (PD-1) and its ligand PD-L1 are shown to be involved in alleviating aGVHD and cGVHD in preclinical studies.^40–42^ Blockade of PD-1/PD-L1 exacerbates aGVHD mortality via augmenting systemic IFN-γ levels in a mouse allo-HSCT model.^40^ In addition, depletion of PD-L1 expression in recipients allowed donor CD8^+^ T cell expansion and accelerated GVHD lethality^41^ whereas inhibition of the PD-1/PD-L1 pathway exacerbated cGVHD in a minor antigen mismatched model.^42^ These studies provide insights into the potential clinical value of exploring PD-1 on T cells in allo-HSCT immune tolerance. However, it remains elusive whether G-CSF-induced PD-1 expression on T cell subsets is relevant to aGVHD. BCL6 (B cell lymphoma 6) was first cloned from diffuse lymphomas with large cell component samples in 1993,^43^ which is a lineage-defining transcription factor for T follicular helper cells (Tfh) and regulates the expression of PD-1 in Tfh.^44–46^ BCL6 is also reported to regulate CD8^+^ T function by maintaining CD8^+^ T cell stemness, suppressing the differentiation of intratumorally exhausted CD8^+^ stem- or progenitor-like cells into terminally differentiated cells.^47,48^ Nevertheless, the role of BCL6 on PD-1 expression in CD8^+^ T cells and immunoregulation of CD8^+^ T cells is yet to be elucidated.

In this study, we comprehensively analyzed the peripheral T lymphocyte subsets from 80 G-CSF mobilized donors and their correlation with the aGVHD occurrence in the 80 corresponding recipients. From this systematic analysis, we identified a novel donor peripheral T_SCM_-like regulatory subset (PD-1^+^CD8^+^CD45RA^+^CCR7^+^) which was significantly positively correlated with alleviated aGVHD and lower infection in recipients. Remarkably, these T cells were T_SCM_-like regulatory cells that possessed dual functions of Treg and Teff because they were able to inhibit aGVHD while simultaneously activating GVL for optimal clinical outcomes in recipients. We further verified the functions of these T_SCM_-like regulatory cells in a mouse allo-HSCT model and showed that the PD-1^+^CD8^+^ T_SCM_-like regulatory subset indeed existed in the peripheral blood and bone marrow of clinical recipients. Finally, we revealed that in both mice and human G-CSF upregulated the PD-1 expression via BCL6 on these T cells. In summary, our clinical association study, immunophenotyping, single-cell genomics and animal model investigation have identified a novel T_SCM_-like regulatory subset that not only can serve as a potentially new clinical cellular marker for haploidentical donor selection but also may be used for cell-based therapy for aGVHD and other auto-immune diseases.

## Results

### G-CSF mobilization expands donor peripheral PD-1^+^CD8^+^CD45RA^+^CCR7^+^ T cell subset

**Fig. 1:**
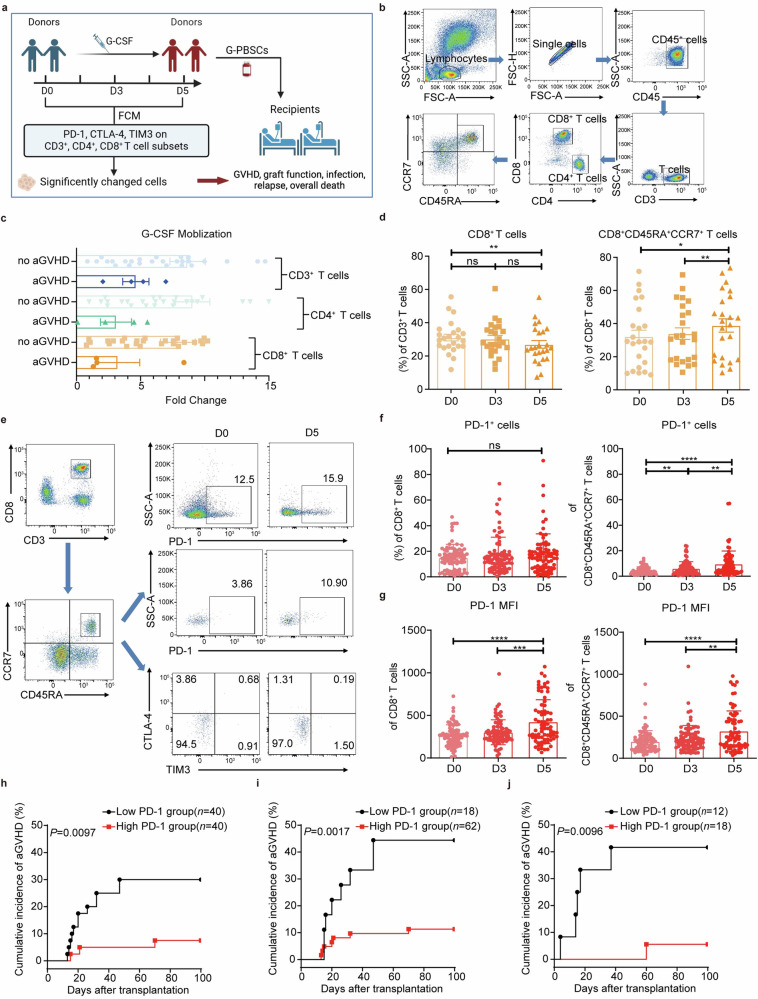
**PD-1**^**+**^**CD8**^**+**^**CD45RA**^**+**^**CCR7**^**+**^ **T cell proportion increases in donor PB after G-CSF mobilization and positively correlates with lower incidence of aGVHD of allogeneic HSCT recipients. a** Outline of clinical experiments and analyses. Donor T cell subsets and inhibitor receptors (IRs) (PD-1, CTLA-4, TIM3) populations in different subsets before and after G-CSF mobilization were analyzed by FCM. Correlation between significantly changed cell proportions and recipients’ outcomes (GVHD, graft function, infection, relapse, overall death) was calculated. The image was created using Biorender (https://biorender.com/). **b** The FCM gating strategy of CD45^+^ cells in donor PB before and after G-CSF mobilization. The CD45^+^ cells were analyzed for the compositions of CD3^+^ T cells, CD4^+^ T cells, CD8^+^ T cells, and CD45RA^+^CCR7^+^ T cells in each subset. **c** Fold change of CD3^+^ (blue), CD4^+^ (green), and CD8^+^ (orange) T cell counts in donor PB corresponding to the recipients with and without aGVHD by FCM (*n* = 33). **d** The percentages of CD8^+^ T cells in total lymphocytes and CD45RA^+^CCR7^+^ T cells in CD8^+^ T cells before (D0) and after three (D3) or five (D5) days of G-CSF mobilization to the healthy donors by FCM (*n* = 24). **e** The FCM gating strategy of analyzing the PB of healthy donors before and after G-CSF mobilization. The PD-1^+^ cells, TIM3^+^ cells and CTLA-4^+^ cells were from CD8^+^CD45RA^+^CCR7^+^ T cell subsets. **f** The percentages of PD-1^+^ cells in the CD8^+^ T cells and CD8^+^CD45RA^+^CCR7^+^ T cells on D0, D3 and D5 of G-CSF mobilization to the healthy donors by FCM (*n* = 80). **g** The MFI of PD-1 in the CD8^+^ T cells and CD8^+^CD45RA^+^CCR7^+^ T cells on D0, D3 and D5 of G-CSF mobilization to the healthy donors by FCM (*n* = 80). **h** Kaplan–Meier analysis of the cumulative incidence of aGVHD stratified by the frequencies of PD-1^+^ cells in donor peripheral CD8^+^CD45RA^+^CCR7^+^ T cells on the fifth day of G-CSF mobilization (D5) (*n* = 80). Recipients were divided into a high PD-1 group (red) and a low PD-1 group (black) according to the median frequencies of PD-1^+^ cells in peripheral CD8^+^CD45RA^+^CCR7^+^ T cells (6.675%) of their donors on D5. **i** Kaplan–Meier analysis of the cumulative incidence of aGVHD stratified by the highest frequencies of PD-1^+^ cells in peripheral CD8^+^CD45RA^+^CCR7^+^ T cells of the relevant donor on the third or fifth day of G-CSF mobilization (D3 or D5) (*n* = 80, discovery set). Recipients were divided into the high PD-1 group (red) and low PD-1 group (black) according to the cut-off value of the highest PD-1^+^ cell frequencies in peripheral CD8^+^CD45RA^+^CCR7^+^ T cells (3.4%) of their donors on D3 or D5. **j** Kaplan–Meier analysis of the cumulative incidence of aGVHD stratified by the highest frequencies of PD-1^+^ cells in peripheral CD8^+^CD45RA^+^CCR7^+^ T cells of the relevant donor on the third or fifth day of G-CSF mobilization (D3 or D5) (*n* = 30, validation set). Recipients were divided into the high PD-1 group (red) and low PD-1 group (black) according to the cut-off value of the highest PD-1^+^ cell frequencies in peripheral CD8^+^CD45RA^+^CCR7^+^ T cells (3.4%) of their donors on D3 or D5. Data are presented as the mean ± SEM, *P < 0.05, **P < 0.01, ***P < 0.001, ****P < 0.0001

To investigate the correlation of recipient aGVHD and specific immune cell subset in donor peripheral blood (PB) after G-CSF mobilization, we enrolled 80 donors and 80 recipients of allo-HSCT and aimed to examine the T cell subsets in healthy donor PB before and after G-CSF mobilization and their correlation with the incidence of recipient aGVHD (Fig. 1a). We first used flow cytometry (FCM) to evaluate the effect of G-CSF mobilization on donor peripheral T cell subsets on the day before mobilization (D0), the third (D3), and the fifth (D5) day after mobilization (Fig. 1b). We found that G-CSF mobilization significantly increased donor peripheral T cell numbers (Fig. 1c). Interestingly, higher donor T cell numbers correlated to lower incidence of recipient aGVHD, indicating that G-CSF may expand certain GVHD-suppressing donor T cells (Fig. 1c). We observed that while total CD8^+^ T cell frequencies slightly decreased on D5, that of CD8^+^CD45RA^+^CCR7^+^ T cell increased significantly (Fig. 1d). On the other hand, there were no changes of donor CD3^+^ and CD4^+^ T cells and their naïve subsets (CD45RA^+^CCR7^+^) upon G-CSF mobilization (Supplementary Fig. 1a, b). Next, we measured the PD-1 expression on donor peripheral CD8^+^ T cell subsets before and after G-CSF mobilization (Fig. 1e). Whereas the proportion of PD-1^+^CD8^+^ T cells did not change, PD-1^+^CD8^+^CD45RA^+^CCR7^+^ T cell frequencies were significantly elevated on D5 (Fig. 1f), which were independent of the donor age and gender (Supplementary Fig. 2a, b). Notably, the increase of G-CSF-induced donor peripheral PD-1^+^CD8^+^CD45RA^+^CCR7^+^ T cells positively correlated with lower recipient aGVHD (Supplementary Fig. 2c, d).

Moreover, both CD8^+^ and CD8^+^CD45RA^+^CCR7^+^ T cell subsets showed higher mean fluorescence intensity (MFI) of PD-1, indicating modulation of PD-1 expression by G-CSF (Fig. 1g). On the other hand, PD-1 expressions of CD3^+^ and CD4^+^ T cells, and their CD45RA^+^CCR7^+^ subsets did not change before and after G-CSF mobilization (Supplementary Fig. 3a–d). In addition, PD-1^+^ cells did not increase in other CD8^+^ T cell subsets (CD45RA^+^CCR7^−^, CD45RA^−^) as significantly as in the CD45RA^+^CCR7^+^ population (Supplementary Fig. 3e, f). CTLA-4 and TIM3 expressions were also detected on total CD3^+^, CD4^+^, and CD8^+^ T cells and corresponding CD45RA^+^CCR7^+^ subsets (Fig. 1e). Despite the decrease of CTLA-4^+^CD4^+^CD45RA^+^CCR7^+^ T cells on D5 (Supplementary Fig. 4), there were no change of CTLA-4^+^ or TIM3^+^ cells in other subsets. We conclude that G-CSF mobilization substantially expands PD-1^+^CD8^+^CD45RA^+^CCR7^+^ T cells in donor PB.

### Donor peripheral PD-1^+^CD8^+^CD45RA^+^CCR7^+^ T cells positively correlate with lower incidence of recipient aGVHD

To explore the potential clinical consequence of donor PD-1^+^CD8^+^CD45RA^+^CCR7^+^ T cells, we evaluated 80 recipients receiving G-PBSC grafts from 80 donors (Supplementary Table 1) and analyzed the potential influence of donor PD-1^+^CD8^+^CD45RA^+^CCR7^+^ T cells on clinical outcomes by stratifying recipients into two groups based on the median percentage (6.675%) of PD-1^+^CD8^+^CD45RA^+^CCR7^+^ T cells on D5 with 40 patients each in the high or low groups. Remarkably, we found that recipients who received high percentages of PD-1^+^CD8^+^CD45RA^+^CCR7^+^ T cells exhibited much lower incidences of aGVHD (Fig. 1h).

To further validate this T cell subset as a clinical indicator for donor selection, we performed ROC analysis and determined the cut-off value of the highest PD-1^+^ cell frequencies in CD8^+^CD45RA^+^CCR7^+^ T cells of donor on D3 or D5: 3.4%, which had the highest Jorden index (sensitivity + specificity -1). We next separated recipients into two groups based on this cut-off value: those grafted with lower percentage of PD-1^+^CD8^+^CD45RA^+^CCR7^+^ T cells (≤3.4%, 18 recipients) and those with higher percentage of this subset (>3.4%, 62 recipients). Similarly to what we found above, recipients who received high percentages of PD-1^+^CD8^+^CD45RA^+^CCR7^+^ T cells exhibited much lower incidences of aGVHD (Fig. 1i). To further verify our findings of the 80-patient discovery cohort, we recruited a 30-patient validation cohort. As expected, higher than cut-off value of donor PD-1^+^CD8^+^CD45RA^+^CCR7^+^ T cells correlated with lower recipient aGVHD (Fig. 1j). Next, we examined the function of this novel T cell subset in both haploidentical and identical transplantation by calculating incidences of 6 types of clinical outcomes (aGVHD, graft failure, infection, cGVHD, relapse, death) in patients stratified by the cut-off value of 3.4% (mean ± SEM, Supplementary Table 2). Interestingly, we found that except graft failure, the incidences of the other 5 outcomes were all lower in the high PD-1 patients than those of the low PD-1 patients. Notably, incidences of aGVHD and infection showed the most significant decrease. We also noticed that the incidences of all 6 clinical outcomes in haploidentical recipients with high PD-1 were all lower than those with low PD-1. They also had significantly lower aGVHD incidence (*P* = 0.0398) (Supplementary Table 2). Among identical recipients, except graft failure and relapse, the incidence of 4 other clinical outcomes in the high PD-1 patients was lower than those with low PD-1 and aGVHD and infection incidences both significantly decreased (Supplementary Table 2). Therefore, the donor PD-1^+^CD8^+^CD45RA^+^CCR7^+^ T cells percentage clearly exhibited a negative correlation with aGVHD occurrence in both haploidentical and identical transplantation. Intriguingly, we also revealed that this subset negatively correlated with recipient infection occurrence post-transplantation.

To further evaluate the donor PD-1^+^CD8^+^CD45RA^+^CCR7^+^ T subset as a donor selection peripheral cellular maker, we stratified 80 donors into four groups according to gender/age (median: 36) and haploidentical or identical transplantations (Supplementary Table 3). We observed that, compared to other demographic groups, young males (36 years old and younger) were the most likely population to have PD-1^+^ cell frequencies higher than the cut-off value (Supplementary Table 3). Specifically, 81.5% of young male donors in all transplantations, 81% in haploidentical transplantation, and 83.3% in identical transplantation had >3.4% peripheral PD-1^+^CD8^+^CD45RA^+^CCR7^+^ T cells. Next, we developed a predictive model to evaluate the sensitivity, specificity, and AUC for donors from each group as an indicator for recipient aGVHD incidence. In line with the percentage of donors (with peripheral PD-1^+^CD8^+^CD45RA^+^CCR7^+^ T cells >3.4%), AUC of the males ≤36 years old group was also the highest (0.67 in all transplantation; 0.69 in haploidentical transplantation) which meanwhile exhibited high sensitivity and specificity (66.7%, 87.6% in all transplantation; 66.7%, 88.9% in haploidentical transplantation) (Supplementary Table 3). On the other hand, concerning our donor cohort, we followed the current clinical guideline that favored young male, which may cause some bias in the sample selection of this study. Nonetheless, our data seemed to agree with current clinical guideline that young male had the highest probability to be favorable donors. And more importantly, we believe that PD-1^+^CD8^+^CD45RA^+^CCR7^+^ T subset may be a bona fide donor selection indicator in addition to the more general demographic indicator of young male.

It should be noted that all graft cells in recipients were originally from donor PB, including the graft PD-1^+^CD8^+^CD45RA^+^CCR7^+^ T subset. Our data fully confirmed the significantly positive correlation between the PD-1^+^CD8^+^CD45RA^+^CCR7^+^ T cell count in the grafts and that of the donor PB (*r* = 0.92, *P* < 0.0001, Supplementary Fig. 5). Therefore, we proposed that using the percentage of this cell subset in donor peripheral blood rather than in the graft is more acquirable to predict the recipient’s prognosis.

### PD-1^+^CD8^+^CD45RA^+^CCR7^+^ T cell subset exhibits T_SCM_-like regulatory features

**Fig. 2:**
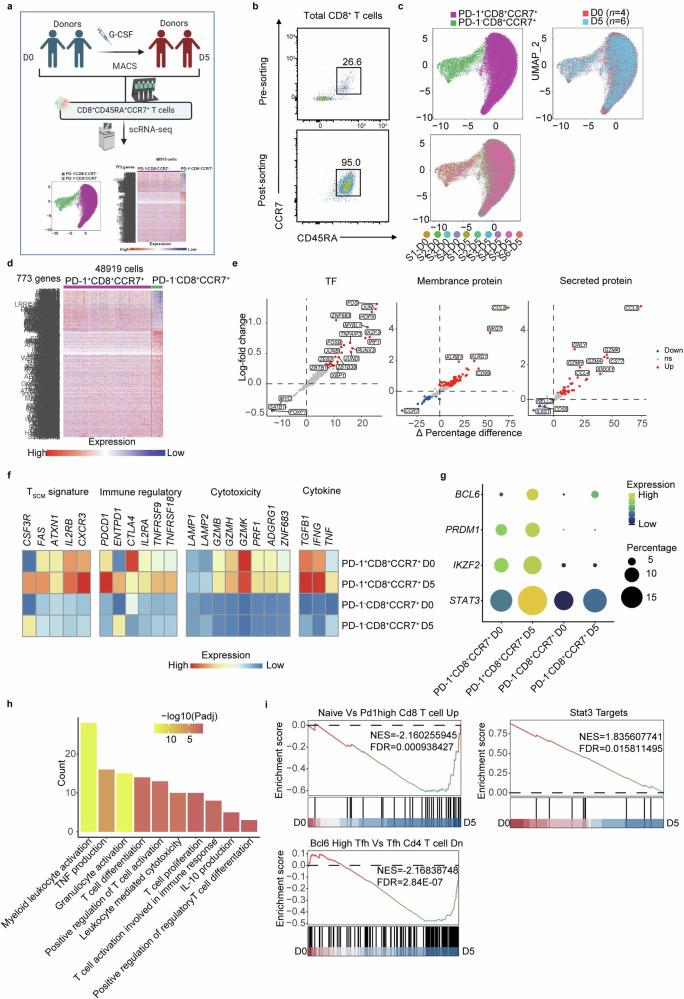
**The PD-1**^**+**^ **subset of CD8**^**+**^**CD45RA**^**+**^**CCR7**^**+**^ **T cells exhibits T**_**SCM**_**-like features and gene expression patterns associated with immune regulatory or cytotoxicity, which are enhanced after G-CSF mobilization. a** Outline shows the workflow of scRNA-seq on CD8^+^CD45RA^+^CCR7^+^ T cells in the PB of healthy donors before (D0) and after (D5) G-CSF mobilization. The image was created using Biorender (https://biorender.com/). **b** Representative plots showing sorted CD8^+^CD45RA^+^CCR7^+^ T cells for scRNA-seq by FCM. **c** UMAP of scRNA-seq data from donor peripheral CD8^+^CD45RA^+^CCR7^+^ T cells before (*n* = 4) or after (*n* = 6) G-CSF mobilized. Cells were grouped into two clusters, including PD-1^−^CD8^+^CCR7^+^ cells and PD-1^+^CD8^+^CCR7^+^ cells. The origins of donors were also presented in UMAP. The cell number of each group had been equalized. **d** Heatmap of the 773 signature genes (215 genes in PD-1^−^CD8^+^CCR7^+^ cells and 558 genes in PD-1^+^CD8^+^CCR7^+^ cells) in the two CD8^+^CD45RA^+^CCR7^+^ clusters. **e** Differential gene expression analysis using the log-fold change expression versus the difference in the percentage of cells expressing the gene between PD-1^+^CD8^+^CCR7^+^ and PD-1^−^CD8^+^CCR7^+^ cells (Δ Percentage difference). Genes with log-fold change >0.3, Δ Percentage difference >10%, and adjusted *P*-value from Wilcoxon test <0.05 were labeled, including TFs associated with AP-1 pathway (*JUN, JUNB, JUND, FOSB, FOS*), genes associated with chemotaxis of immune cells (*CCL5, CCL4*) and cytotoxicity (*GZMB, GZMK, GZMH, GNLY*). **f** The distinct expressions of selected genes associated with G-CSF receptor (*CSF3R*), T_SCM_ signature (*FAS, ATXN1, IL2RB, CXCR3*), immune regulatory (*PDCD1, ENTPD1, CTLA4, IL2RA, TNFRSF9, TNFRSF18*), cytotoxicity (*LAMP1, LAMP2, GZMB, GZMK, GZMH, PRF1, ADGRG1, ZNF683*), and cytokines (*TGFB1, IFNG*, *TNF*) in PD-1^−^CD8^+^CCR7^+^ cells and PD-1^+^CD8^+^CCR7^+^ cells of D0 and D5 were displayed by heatmap. **g** Dotplot presented the expressions of select transcription factors (*BCL6, PRDM1, IKZF2, STAT3*) in PD-1^−^CD8^+^CCR7^+^ cells and PD-1^+^CD8^+^CCR7^+^ cells of D0 and D5. **h** Selected gene ontology terms, including the pathway associated with T cell differentiation, immune regulation (IL-10 production, positive regulation of regulatory T cell differentiation), cytotoxicity (TNF production, leukocyte mediated cytotoxicity, cell killing), and proliferation (positive regulation of T cell activation, T cell activation involved in immune response, T cell proliferation), were enriched in PD-1^+^CD8^+^CCR7^+^ cells of D5 compared with their D0 equivalents. Bars represented the gene ontology terms by a discrete color scale with the count of enriched genes. Bar length represented the −log_10_ (adjusted *P*-value) of the gene ontology terms. **i** GSEA for comparing the enrichment of differentially expressed genes between PD-1^+^CD8^+^CCR7^+^ cells of D0 and D5 in the genes upregulated in comparison of CD8^+^ T_N_ versus PD-1 high CD8 T cells (accession GEO: GSE26495), genes down-regulated in BCL6 high follicular helper T cells (Tfh) versus all Tfh (accession GEO: GSE24574), and genes upregulated in RWPE-1 cells by activated STAT3^103^

We next used scRNA-seq to investigate the molecular features of this T cell subset pre- (D0) and post-G-CSF (D5) mobilization. We purified total CD8^+^CD45RA^+^CCR7^+^ T cells from donors for scRNA-seq and obtained high-quality data including D0 and D5 (Fig. 2a, b). Uniform manifold approximation and projection (UMAP) analysis revealed that total CD8^+^CD45RA^+^CCR7^+^ T cells can be grouped into two clusters: PD-1^+^CD8^+^CCR7^+^ T cells and PD-1^−^CD8^+^CCR7^+^ T cells (Fig. 2c). The purity of CD8^+^CD45RA^+^CCR7^+^ T cells was confirmed by the expressions of signature genes (Supplementary Fig. 6a).

To define characteristics and differences of the two clusters, we examined the differentially expressed genes (Fig. 2d) and focused on genes coding transcription factor (TF), membrane protein and secreted protein. We found that expressions of genes coding TFs associated with AP-1 pathway were upregulated in PD-1^+^CD8^+^CCR7^+^ T cells, along with genes associated with chemotaxis of immune cells and cytotoxicity (Fig. 2e). Similar analysis on the two clusters before and after G-CSF mobilization showed increased expressions of TFs that bind the promoters of G-CSF receptor genes as well as genes associated with host defense on D5 (Supplementary Fig. 6b–e).^49^ Interestingly, PD-1^+^CD8^+^CCR7^+^ T cells not only appeared to have a T_SCM_-like gene expression pattern (*CD27*^+^*CD28*^+^*FAS*^+^*CXCR3*^+^*ATXN1*^+^*IL2RB*^+^)^50^ with increased PD-1 expression, but also expressed lower levels of naïve T cell (T_N_) signature genes, whereas PD-1^−^CD8^+^CCR7^+^ T cells highly expressed T_N_ markers, including *LEF1*, *SELL*, and *IL7R* (Fig. 2f and Supplementary Fig. 6a). Furthermore, PD-1^+^CD8^+^CCR7^+^ T cells on D5 showed higher expressions of T_SCM_ signature genes (*FAS, CXCR3, IL2RB*) than on D0 (Fig. 2f), indicating the effect of G-CSF mobilization on promoting the T_SCM_-like phenotype. G-CSF mobilization also elevated the expression of *CSF3R* which codes G-CSFR in both subsets, suggesting a positive feedback of the G-CSF pathway (Fig. 2f). In addition, compared to PD-1^−^CD8^+^CCR7^+^ T cells, PD-1^+^CD8^+^CCR7^+^ T cells expressed higher levels of genes related to immune regulation, cytotoxicity (Fig. 2f),^51^ and those coding cytokines associated with immunomodulation and cytotoxicity (Fig. 2f). Interestingly, the expressions of these genes is further increased in PD-1^+^CD8^+^CCR7^+^ T cells upon G-CSF treatment (Fig. 2f). Taken together, PD-1^+^CD8^+^CCR7^+^ T cells possessed both immunomodulation and cytotoxic capacity.

In addition, we found that expressions of TFs related to the G-CSF pathway (*STAT3*, *BCL6*, *PRDM1*), and the TF associated with Treg function (*IKZF2*), were all increased in PD-1^+^CD8^+^CCR7^+^ T cells on D0 and D5 (Fig. 2g). Consistently, expressions of these genes were higher in PD-1^+^CD8^+^CCR7^+^ T cells after G-CSF treatment (Fig. 2g). Given that STAT3 regulates BCL6 expression^52,53^ and BCL6 positively regulates PD-1 expression,^45^ we speculated that G-CSF may regulate PD-1 via the STAT3-BCL6 axis.

We next compared gene expression signatures of the PD-1^+^CD8^+^CCR7^+^ and PD-1^−^CD8^+^CCR7^+^ subsets on D0 and D5 by gene ontology (GO) analysis and found notable difference between the two subsets (Supplementary files of GO analysis). Pathways enriched in the PD-1^+^CD8^+^CCR7^+^ T cells included the activation of immune response, cell killing, IκB kinase/NFκB signaling, leukocyte proliferation, while those enriched in PD-1^−^CD8^+^CCR7^+^ T cells were mostly ribosome-associated pathways (ribosome biogenesis, ribonucleoprotein complex biogenesis). PD-1^+^CD8^+^CCR7^+^ T cells on D5 were also enriched with myeloid leukocyte activation and granulocyte activation, possibly reflecting the influence of G-CSF mobilization (Fig. 2h). Similarly, the pathway associated with T cell differentiation, immune regulation, cytotoxicity, and proliferation were also enriched upon G-CSF mobilization (Fig. 2h). The myeloid leukocyte activation and granulocyte activation pathway were also enriched in PD-1^−^CD8^+^CCR7^+^ T cells, indicating the unbiased effect of G-CSF on both clusters (Supplementary files of GSEA analysis).

Gene set enrichment analysis (GSEA) further revealed that highly expressed genes in PD-1^+^CD8^+^CCR7^+^ T cells were featured genes of PD-1^high^ CD8^+^ T cells and leukocyte proliferation, whereas those of PD-1^−^CD8^+^CCR7^+^ T cells were mostly CD8 T_N_ specific (Supplementary Fig. 7a). Genes enriched in PD-1^+^CD8^+^CCR7^+^ T cells on D5 were mostly signature genes of PD-1^high^CD8^+^ T cells, BCL6^high^ Tfh or targeted by STAT3, whereas those of D0 were similar to genes featured in CD8 T_N_ and BCL6^−^ Tfh cells (Fig. 2i). On the other hand, the gene expression pattern in PD-1^−^CD8^+^CCR7^+^ T cells on D5 was more similar to that of the bone marrow neutrophil than those on D0 (Supplementary Fig. 7b). Altogether, scRNA-seq analysis reveals that G-CSF mobilization induced a unique gene expression signature in PD-1^+^CD8^+^CCR7^+^ cells.

### G-CSF-induced PD-1^+^CD8^+^CD45RA^+^CCR7^+^ T cells express both Treg and Teff markers

To further verify the T_SCM_-like feature of the PD-1^+^CD8^+^CD45RA^+^CCR7^+^ T cell subset that exhibits both immune regulatory and cytotoxicity phenotype, we used FCM to measure the protein expressions of key molecules identified in scRNA-seq.

Given that PD-1 is a marker for CD8^+^ Treg cells^54–59^ and a subtype of CD8^+^CD45RA^+^CCR7^+^ T cells in PB may represent a novel immunosuppressive CD8^+^ T cell subset,^60^ we wanted to further define the immunophenotypes of the PD-1^+^CD8^+^CD45RA^+^CCR7^+^ T cell subset. Based on the transcriptome feature revealed by scRNA-seq (Fig. 2), we speculated that the G-CSF-induced PD-1^+^CD8^+^CD45RA^+^CCR7^+^ T cells may be a critical regulatory CD8^+^ T subset that alleviates aGVHD.

**Fig. 3:**
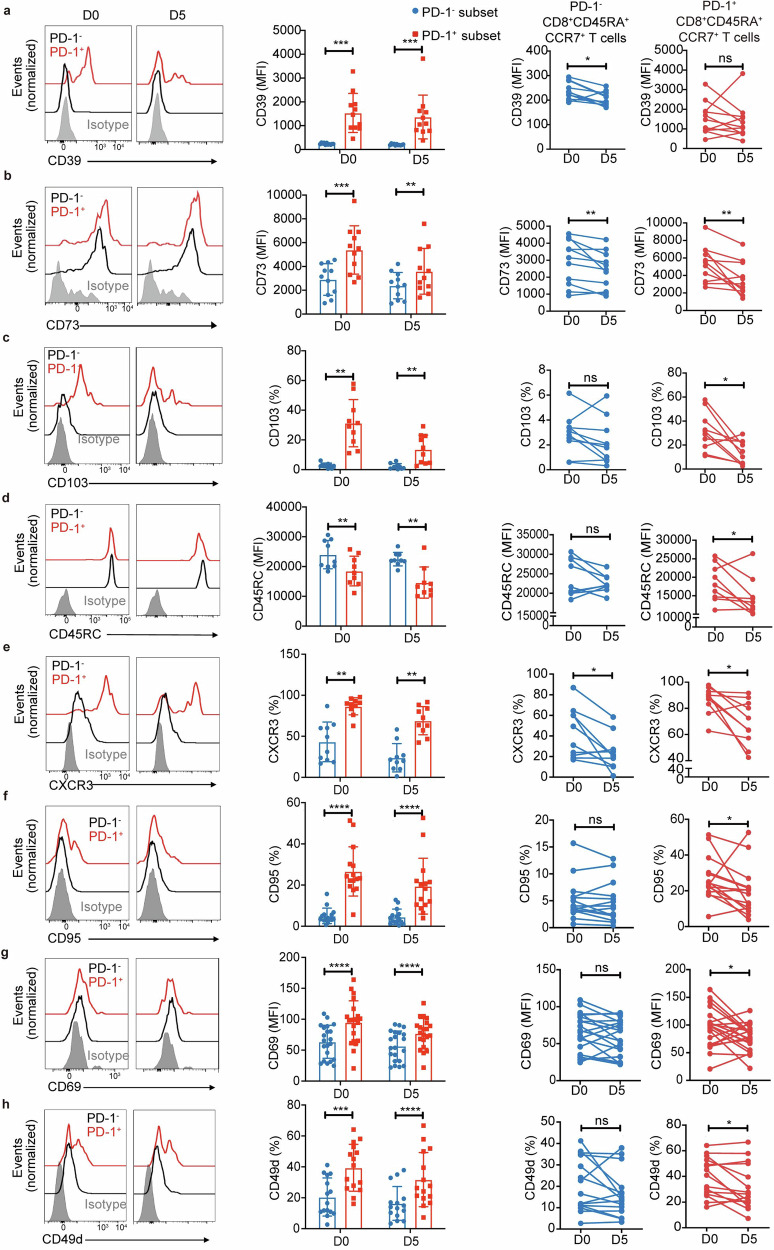
**G-CSF-induced PD-1**^**+**^**CD8**^**+**^**CD45RA**^**+**^**CCR7**^**+**^ **T cells exhibit both Treg and effector functional markers. a**, **b** Representative plots and the MFI of CD39 (**a**) and CD73 (**b**) in PD-1^−^ (black/blue) or PD-1^+^ (red) CD8^+^CD45RA^+^ CCR7^+^ T cells identified by FCM in PB of healthy donors before (D0) and after G-CSF mobilization for five days (D5) by FCM (CD39: *n* = 11; CD73: *n* = 11). **c** Representative plots and the percentages of CD103^+^ cells in PD-1^−^ (black/blue) or PD-1^+^ (red) CD8^+^CD45RA^+^ CCR7^+^ T cells in the PB of healthy donors in D0 and D5 by FCM (*n* = 10). **d** Representative plots and the MFI of CD45RC in PD-1^−^ (black/blue) or PD-1^+^ (red) CD8^+^CD45RA^+^ CCR7^+^ T cells in PB of healthy donors in D0 and D5 by FCM (*n* = 9). **e**, **f** Representative plots and the percentages of CXCR3^+^ (**e**) and CD95^+^ (**f**) cells in PD-1^−^ (black/blue) or PD-1^+^ (red) CD8^+^CD45RA^+^ CCR7^+^ T cells in PB of healthy donors in D0 and D5 by FCM (CXCR3: *n* = 10; CD95: *n* = 15). **g** Representative plots and the MFI of CD69 in PD-1^−^ (black/blue) or PD-1^+^ (red) CD8^+^CD45RA^+^ CCR7^+^ T cells in PB of healthy donors in D0 and D5 by FCM (*n* = 21). **h** Representative plots and the percentages of CD49d in PD-1^−^ (black/blue) or PD-1^+^ (red) CD8^+^CD45RA^+^CCR7^+^ T cells in PB of healthy donors in D0 and D5 by FCM (*n* = 15). Data are presented as the mean ± SEM, *P < 0.05, **P < 0.01, ***P < 0.001, ****P < 0.0001

We analyzed featured functional markers of CD8^+^ Treg or CD8^+^ Teff and compared their expression in PD-1^+^CD8^+^CD45RA^+^CCR7^+^ T and PD-1^−^CD8^+^CD45RA^+^CCR7^+^ T cells. PD-1^+^CD8^+^CD45RA^+^CCR7^+^ T cells expressed higher levels of CD39 in both pre- and post-G-CSF mobilization (Fig. 3a), consistently with the scRNA-seq results (Fig. 2f). Moreover, CD73 and CD103 also showed higher expression (Fig. 3b, c) like CD8^+^ Treg.^58,61,62^ CD8^+^ Treg expressed low CD45RC.^58,63–65^ The PD-1^+^ subset also expressed significantly lower CD45RC in both pre- and post-G-CSF mobilization compared to the PD-1^−^ subset (Fig. 3d).

Remarkably, PD-1^+^CD8^+^CD45RA^+^CCR7^+^ T cells also exhibited increased expression of CD8^+^ Teff markers, including CXCR3, CD95 (also a T_SCM_ maker) and consistently with the scRNA-seq results (Fig. 2f), CD69 (also a T_SCM_ maker) and CD49d, both pre- and post-G-CSF mobilization (Fig. 3e–h). Notably, G-CSF mobilization slightly reduced the expressions of these markers though their expressions remained appreciable. Co-stimulatory molecules CD28, CD127, and CD62L did not show substantial expression differences between PD-1^+^CD8^+^CD45RA^+^CCR7^+^ and PD-1^−^CD8^+^CD45RA^+^CCR7^+^ subsets (Supplementary Fig. 8a–c) which indicated that both subsets exhibited a non-terminally differentiated phenotype.

Our results demonstrate that PD-1^+^CD8^+^CD45RA^+^CCR7^+^ T cells are unique in that they exhibit functional features of both CD8^+^ Treg and CD8^+^ Teff which may be beneficial in the process of allo-HSCT.

### PD-1^+^CD8^+^CD45RA^+^CCR7^+^ T cells have dual functions in preventing GVHD while preserving the GVL effect

**Fig. 4:**
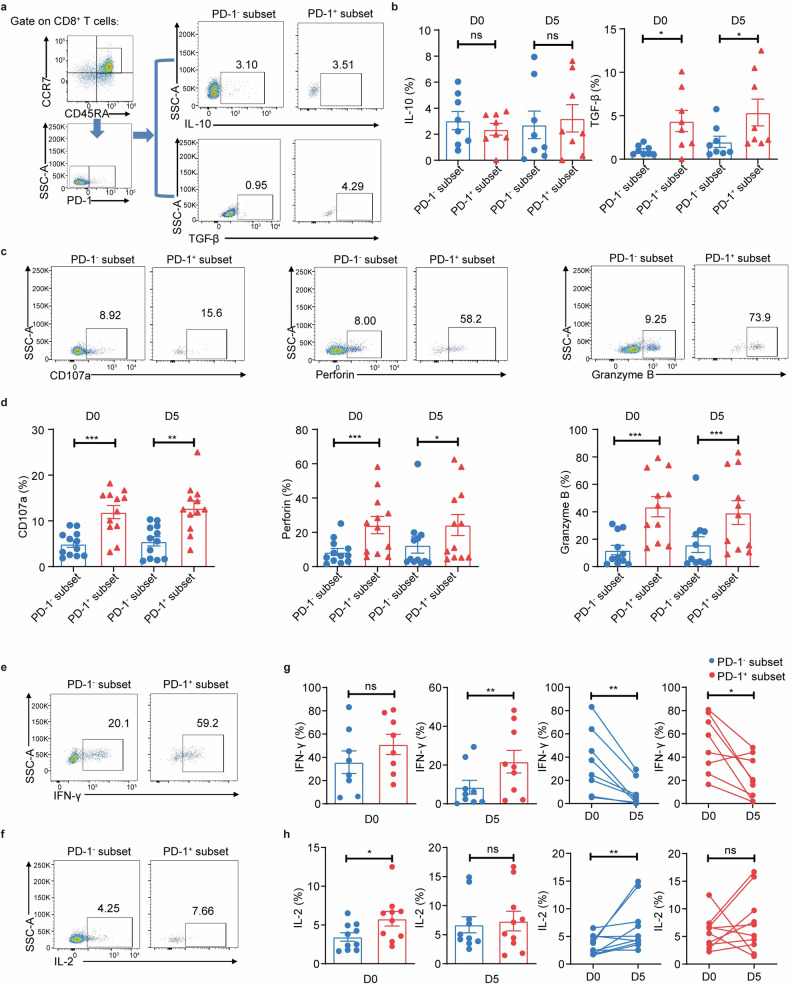
**The PD-1**^**+**^**CD8**^**+**^**CD45RA**^**+**^ **CCR7**^**+**^ **T cells have both regulatory and cytotoxicity T cell characteristics. a**–**h** PBMCs from healthy donors before (D0) and after G-CSF mobilization for five days (D5) were cultured with a cell stimulation cocktail (plus protein transport inhibitors) for 4 h. **a** Representative plots of the expression of IL-10 and TGF-β from CD8^+^CD45RA^+^CCR7^+^ T cell subsets (PD-1^−^ and PD-1^+^, blue and red) of D0. **b** The percentages of IL-10^+^ and TGF-β^+^ cells in PD-1^−^ (blue) or PD-1^+^ (red) CD8^+^CD45RA^+^CCR7^+^ T cells in PB of D0 and D5 by FCM (*n* = 8). **c** Representative plots of CD107a, Perforin, and Granzyme B on the CD8^+^CD45RA^+^CCR7^+^ T cell subsets (PD-1^−^ and PD-1^+^, blue and red). **d** The percentages of CD107a^+^, Perforin^+,^ and Granzyme B^+^ cells in PD-1^−^ (blue) or PD-1^+^ (red) CD8^+^CD45RA^+^CCR7^+^ T cells in PB of D0 and D5 by FCM (*n* = 11-12). **e**, **f** Representative plots detecting IFN-γ and IL-2 on the CD8^+^CD45RA^+^CCR7^+^ T cell subsets (PD-1^−^ and PD-1^+^). **g**, **h** The percentages of IFN-γ^+^ (*n* = 8) and IL-2^+^ (*n* = 10) cells in PD-1^−^ (blue) or PD-1^+^ (red) CD8^+^CD45RA^+^CCR7^+^ T cells in PB of D0 and D5 by FCM. Data are presented as the mean ± SEM, *P < 0.05, **P < 0.01, ***P < 0.001

Given that PD-1^+^CD8^+^CD45RA^+^CCR7^+^ T cells possess both immunoregulatory and effector gene expression signatures, they may play a unique role in alleviating aGVHD in allo-HSCT. Therefore, we wanted to analyze in detail their cellular functions in both pre- and post-G-CSF mobilization. We first measured the release of immunosuppressive cytokines IL-10 and TGF-β and found that PD-1^+^CD8^+^CD45RA^+^CCR7^+^ T cells expressed higher TGF-β upon G-CSF stimulation, whereas no substantial IL-10 changes were detected (Fig. 4a, b). This was consistent with the scRNA-seq results (Fig. 2f, h), and supported the immune-suppressive activities of PD-1^+^CD8^+^CD45RA^+^CCR7^+^ T cells.

We subsequently investigated proteins involved in cytotoxic activity, including CD107a, perforin, granzyme B, IFN-γ and IL-2. PD-1^+^CD8^+^CD45RA^+^CCR7^+^ subset expressed significantly more CD107a, perforin, and granzyme B both pre- and post-G-CSF mobilization (Figs. 4c, d, 2f). We observed no difference of IFN-γ production on D0. Both subsets had significantly reduced IFN-γ secretion after G-CSF mobilization, with that of PD-1^+^CD8^+^CD45RA^+^CCR7^+^ subset slightly higher. PD-1^+^CD8^+^CD45RA^+^CCR7^+^ cells had higher IL-2 secretion on D0, but the difference became insignificant on D5 (Fig. 4e–h).

**Fig. 5:**
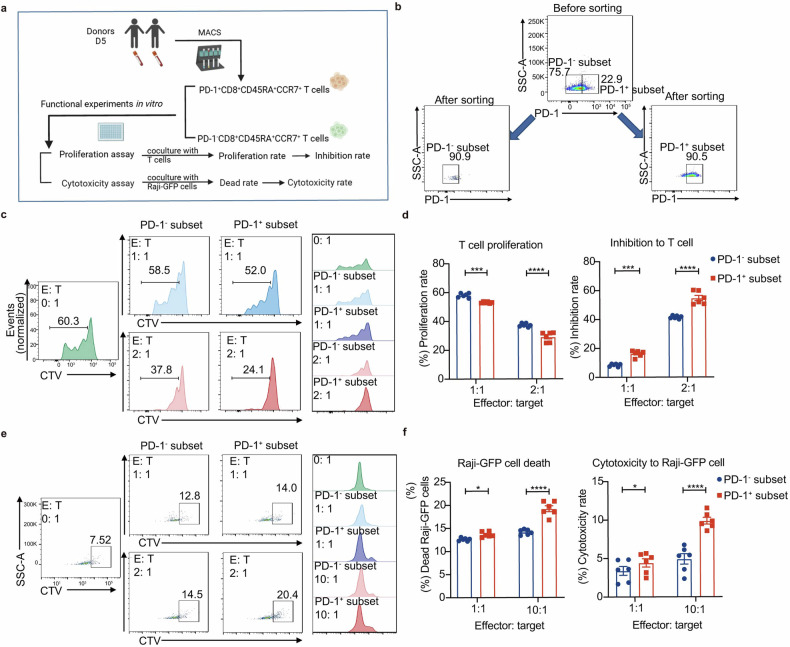
**The PD-1**^**+**^**CD8**^**+**^**CD45RA**^**+**^**CCR7**^**+**^ **T cells have both regulatory and cytotoxicity T cell functions. a** Outline shows the workflow of PD-1^+^CD8^+^CD45RA^+^ CCR7^+^ T cell functional experiments in vitro in the PB of healthy donors after (D5) G-CSF mobilization. The image was created using Biorender (https://biorender.com/). **b** Representative plots showing sorted PD-1^+^CD8^+^CD45RA^+^CCR7^+^ T cells and PD-1^−^CD8^+^CD45RA^+^CCR7^+^ T cells for functional experiments by FCM. **c** Representative plots of proliferating T cells cocultured with PD-1^+^CD8^+^CD45RA^+^CCR7^+^ T cells or PD-1^−^CD8^+^CD45RA^+^CCR7^+^ T cells of healthy donors on D5 in different effector-to-target (E:T) ratios by FCM. **d** Proliferation rate of T cells cocultured with PD-1^+^CD8^+^CD45RA^+^CCR7^+^ T cells or PD-1^−^CD8^+^CD45RA^+^CCR7^+^ T cells of healthy donors on D5 in different effector-to-target (E:T) ratios for 3 days and inhibition rate of PD-1^+^CD8^+^CD45RA^+^CCR7^+^ T cells and PD-1^−^CD8^+^CD45RA^+^CCR7^+^ T cells by FCM (*n* = 6). **e** Representative plots of Raji-GFP cells cocultured with PD-1^+^CD8^+^CD45RA^+^CCR7^+^ T cells or PD-1^−^CD8^+^CD45RA^+^CCR7^+^ T cells of healthy donors on D5 in different effector-to-target (E:T) ratios by FCM. **f** Dead rate of Raji-GFP cell cocultured with PD-1^+^CD8^+^CD45RA^+^CCR7^+^ T cells or PD-1^−^CD8^+^CD45RA^+^CCR7^+^ T cells of healthy donors on D5 in different effector-to-target (E:T) ratios for 4 h and cytotoxicity rate of PD-1^+^CD8^+^CD45RA^+^CCR7^+^ T cells and PD-1^−^CD8^+^CD45RA^+^CCR7^+^ T cells by FCM (*n* = 6). Data are presented as the mean ± SEM, *P < 0.05, ***P < 0.001, ****P < 0.0001

To verify the function of PD-1^+^CD8^+^CD45RA^+^CCR7^+^ subset, we sorted PD-1^+^CD8^+^CD45RA^+^CCR7^+^ and PD-1^−^CD8^+^CD45RA^+^CCR7^+^ subset in donor PB on D5 for in vitro proliferation and cytotoxicity assays (Fig. 5a, b). Results showed that PD-1^+^CD8^+^CD45RA^+^CCR7^+^ subset had a stronger inhibitory effect on homologous T cell proliferation (Fig. 5c, d) and cytotoxicity on Raji cells (Fig. 5e, f).

These results collectively support our conclusion that PD-1^+^CD8^+^CD45RA^+^CCR7^+^ T cells possess both immune modulation and toxicity capacities which leads to both alleviated aGVHD and retained cytotoxic activity for GVL. We define these T cells as PD-1^+^CD8^+^ T_SCM_-like regulatory cells.

### G-CSF-expanded splenic PD-1^+^CD8^+^ T_SCM_-like regulatory cells reduced aGVHD in a mouse allogeneic model and PD-1 is essential for the effect

To verify the function and mechanism of PD-1^+^CD8^+^ T_SCM_-like regulatory cells in vivo, we investigated the effect of G-CSF-induced T cells on aGVHD in a mouse model. We focused on CD8^+^CD44^−^CD62L^+^ T cells, which represent the equivalent of human CD8^+^CD45RA^+^CCR7^+^ T cells.^66^ C57BL/6J donor mice were injected with rhG-CSF to mimic the clinical donor mobilization (Supplementary Fig. 9a).^30^ G-CSF mobilization significantly increased WBC counts in donor PB and spleen, but not bone marrow (BM) (Supplementary Fig. 9b). Furthermore, the frequencies of splenic CD44^−^CD62L^+^ cells were significantly higher in the G-CSF-treated mice both in CD4^+^ and CD8^+^ T cells (Supplementary Fig. 9c). Surprisingly, in the PB, although the WBC number was higher in the G-CSF group, CD44^−^CD62L^+^ cells in PB decreased sharply in both CD4^+^ and CD8^+^ T cells, in comparison to the saline group (Supplementary Fig. 9d). We next analyzed PD-1 expression in the CD44^−^CD62L^+^ T cells after G-CSF mobilization. The PD-1^+^CD44^−^CD62L^+^ cells in the spleen, but not in the PB, were significantly increased both in CD4^+^ and CD8^+^ T cells (Supplementary Fig. 9e, f). Therefore, G-CSF expanded splenic PD-1^+^CD8^+^ T_SCM_-like regulatory cells in mice.

**Fig. 6:**
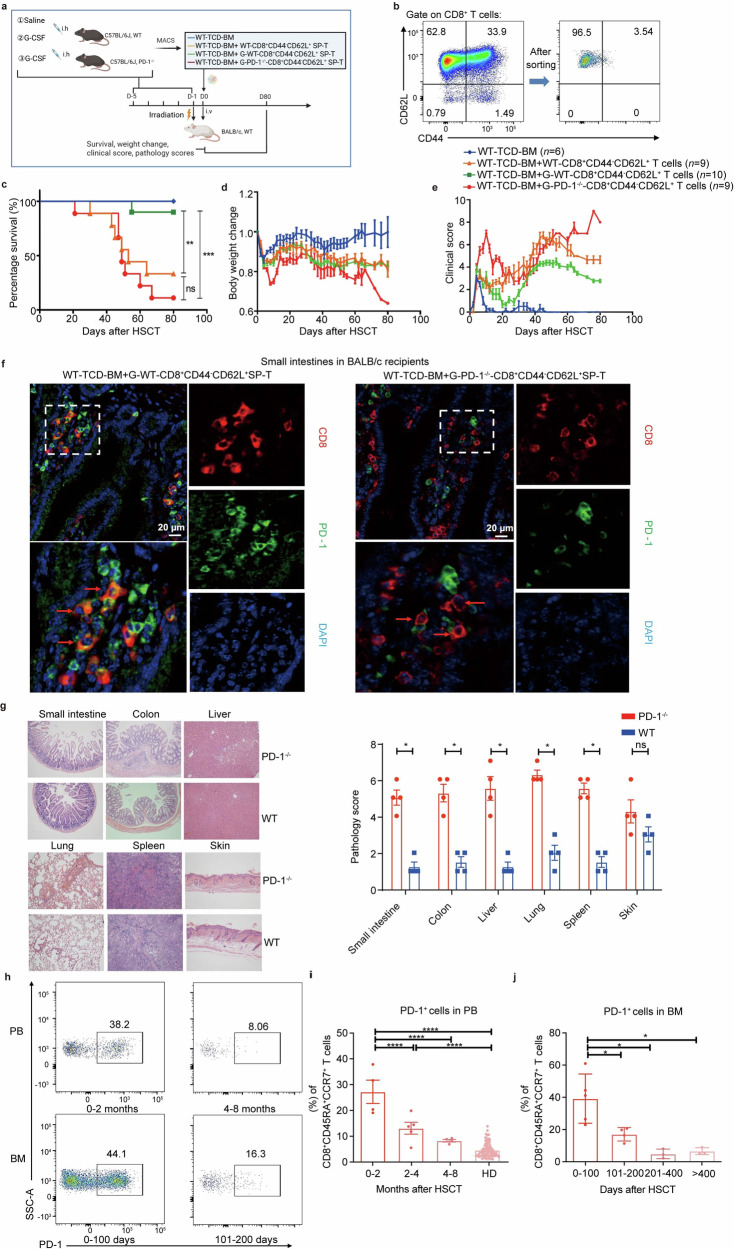
**G-CSF-induced PD-1**^**+**^**CD8**^**+**^ **T**_**SCM**_**-like regulatory cells alleviate aGVHD in mouse allogeneic HSCT model. a** Outline of the mouse allogeneic (MHC-mismatched, C57BL/6J to BALB/c) HSCT model and experiments to evaluate the function of specific T cell subsets. Donor cells: (1) T cell-depleted bone marrow (TCD-BM) cells (5 × 10^6^) of C57BL/6J WT mice (blue); (2) TCD-BM cells plus 5 × 10^5^ splenic CD8^+^CD44^−^CD62L^+^ T cells from C57BL/6J WT mice (saline-treated) (yellow); (3) TCD-BM cells plus 5 × 10^5^ splenic CD8^+^CD44^−^CD62L^+^ T cells from C57BL/6J WT mice (G-CSF-treated) (green); (4) TCD-BM cells plus 5 × 10^5^ splenic CD8^+^CD44^−^CD62L^+^ T cells from PD-1-knockout mice (G-CSF-treated) (red). Lethally irradiated (8 Gy on D-1) BALB/c mice were used as the recipients. The image was created using Biorender (https://biorender.com/). **b** Splenic CD8^+^CD44^−^CD62L^+^ T cells for transplantation were sorted using MACS. **c**–**e** The survival rate (**c**), the body weight change (**d**), and the clinical score (**e**) of recipients were monitored for 80 days after transplantation. **f** Immunofluorescence staining for CD8 (red) and PD-1 (green) of small intestine tissues of BALB/c recipients receiving G-CSF-treated splenic CD8^+^CD44^−^CD62L^+^ T cells from C57BL/6J WT or PD-1^−^^/^^−^ mice described in (**a**). The tissues were collected at the onset of aGVHD on day 47 post allo-HSCT. Nuclei were depicted in blue (4ʹ, 6-diamidino-2-phenylindole, DAPI). Scale bars, 20 μm. **g** Representative hematoxylin and eosin staining images and pathology scores (bar chart on the right) of the BALB/c recipients that received G-CSF-treated splenic CD8^+^CD44^−^CD62L^+^ T cells from either C57BL/6J WT (blue) or PD-1^−^^/^^−^ (red) mice. Tissues were harvested on day 47 after allo-HSCT for analysis (original magnification ×100). **h**–**j** PD-1^+^CD8^+^CD45RA^+^CCR7^+^ cells exist in recipients’ PB and bone marrow (BM). **h** Representative plots of PD-1^+^CD8^+^CD45RA^+^ CCR7^+^ T cells in PB (*n* = 13) and BM (*n* = 15) of recipients by FCM. **i**–**j** The percentages of PD-1^+^ cells in the CD8^+^CD45RA^+^CCR7^+^ T cells in PB (*n* = 13; healthy donors (HD): *n* = 80) and BM (*n* = 15) of recipients by FCM. The recipients’ PB and bone marrow cells were obtained from HSCT hospitalized and follow-up recipients in Ruijin Hospital. Data are presented as the mean ± SEM, *P < 0.05, **P < 0.01, ***P < 0.001, ****P < 0.0001

To further explore the effect of G-CSF-induced PD-1^+^CD8^+^ T_SCM_-like regulatory cells on aGVHD, we established an allogeneic aGVHD model by intravenously (i.v) injecting T cell-depleted bone marrow (TCD-BM) of C57BL/6J mice, with or without splenic CD8^+^CD44^−^CD62L^+^ cells to irradiated BALB/c recipients (Fig. 6a, b). As expected, BALB/c mice receiving C57BL/6J donor TCD-BM alone did not develop aGVHD, whereas most BALB/c mice receiving C57BL/6J donor TCD-BM plus saline-treated CD8^+^CD44^−^CD62L^+^ T cells died of aGVHD (Fig. 6c). We next harvested splenic CD8^+^CD44^−^CD62L^+^ T cells after G-CSF mobilization and repeated the transplantation. Remarkably, CD8^+^CD44^−^CD62L^+^ T cells from G-CSF-treated donor mice substantially improved recipient survival rate and reduced weight loss and clinical scores (Fig. 6c–e). We conclude that CD8^+^CD44^−^CD62L^+^ T subset from G-CSF-treated mice with higher proportions of PD-1^+^CD8^+^ T_SCM_-like regulatory cells exhibit an immunomodulatory effect in allogeneic transplantation.

To investigate the role of PD-1 in the immunoregulation ability of PD-1^+^CD8^+^ T_SCM_-like regulatory cells, we harvested donor T cells from G-CSF-treated PD-1^−^^/^^−^ mice for allogeneic transplantation. In contrast to CD8^+^CD44^−^CD62L^+^ T subset from G-CSF-treated WT mice, the same population of T cells from G-CSF-treated PD-1^−^^/^^−^ mice caused aGVHD, which appeared more severe than using cell graft from saline-treated WT mice (Fig. 6c) and was accompanied by substantially more weight loss and higher clinical scores (Fig. 6d, e).

We next performed a pathological examination of BALB/c recipients that received donor CD8^+^CD44^−^CD62L^+^ T cell grafts from either G-CSF-treated WT or PD-1^−^^/^^−^ mice followed by immunofluorescence of several aGVHD target organs on the 47th days after transplantation. We observed the absence of PD-1 in most CD8^+^ T cells (presumably donor cells) in the small intestine when donor T cells were isolated from PD-1^−^^/^^−^ mice, whereas a considerable amount of CD8^+^ T cells expressed PD-1 in mice engrafted with WT donor T cells (Fig. 6f), indicating homing of both WT and PD-1^−^^/^^−^ T cells in the small intestine. Similar results were also observed in the colon (Supplementary Fig. 10a) and liver (Supplementary Fig. 10b). H&E staining confirmed that relatively less pathological damage in GVHD target organs in recipients transplanted with WT G-CSF-treated donor CD8^+^CD44^−^CD62L^+^ T cells, whereas G-CSF-treated donor PD-1^−^^/^^−^ cells caused more severe damages (Fig. 6g). Notably, PD-1 knockout abolished the immunomodulatory effect of G-CSF-induced T cell subset. Therefore, similar to human donors, PD-1 also marks donor CD8^+^ T_SCM_-like regulatory cells with immunomodulatory effect to alleviate aGVHD in mice.

### G-CSF-expanded splenic PD-1^+^CD8^+^ T_SCM_-like regulatory cells retained GVL in a mouse allogeneic model

Our scRNA-seq, FCM analysis and in vitro function assays all showed that donor G-CSF mobilized PD-1^+^CD8^+^ T_SCM_-like regulatory cells have cytotoxicity phenotype which may lead to GVL effect. To this end, we established a mouse GVL model (Supplementary Fig. 11a) by infusing mouse reticulum cell sarcoma A20 cell to explore the GVL effect exerted by the PD-1^+^CD8^+^ T_SCM_-like regulatory cells.^67^ Briefly, recipients mice were divided into 3 groups and received WT TCD-BM along with 5 × 10^5^ A20 cells; WT TCD-BM, 5 × 10^5^ A20 and WT CD8^+^CD44^−^CD62L^+^ T cells; WT TCD-BM, 5 × 10^5^ A20 and G-CSF-treated WT CD8^+^CD44^−^CD62L^+^ T cells. The recipient mice were monitored every 4 days post-transplantation to assess survival, weight change and peripheral blood A20 cell level. We observed that mice injected with WT TCD-BM and A20 cells died within two weeks after transplantation (Supplementary Fig. 11b). Intriguingly, treatments of CD8^+^CD44^−^CD62L^+^ T cells from either WT or G-CSF-treated WT mice significantly prolonged the survival and inhibited the weight loss of recipient mice (Supplementary Fig. 11b, c). Consistently, we observed that treatment of CD8^+^CD44^−^CD62L^+^ T cells significantly decreased the percentage of A20 cells in the PB of recipient mice 14 days post-transplantation (Supplementary Fig. 11d, e). Of note, G-CSF mobilized CD8^+^CD44^−^CD62L^+^ T cells elicited a better GVL activity given that the percentage of A20 cells in peripheral blood of recipient mice was even more decreased (Supplementary Fig. 11d, e). Taken together, these results indicate that CD8^+^CD44^−^CD62L^+^ T cells from G-CSF-treated mice (with an increased proportion of PD-1^+^CD8^+^CD44^−^CD62L^+^ T cells) in grafts are able to elicit GVL effect in recipient mice.

We next examined whether there was indeed a PD-1^+^CD8^+^ T_SCM_-like regulatory subset in recipient PB and bone marrow (Fig. 6h). To this end, we further analyzed the proportion of PD-1^+^CD8^+^ T_SCM_-like regulatory subset at different time points after HSCT in recipients, as well as in donor PB. The results revealed that in both bone marrow and PB, the proportion of these cells was higher in early post-transplantation. Then the level of these cells decreased, but they remained in recipient weeks and even months after transplantation (mean ± SEM: 27.45 ± 4.580, 13.11 ± 2.348, 8.215 ± 0.5212 respectively 0–2, 2–4, 4–8 months after HSCT in PB, Fig. 6i; 38.94 ± 6.807, 16.90 ± 2.104, 4.737 ± 2.922, 6.430 ± 1.214 respectively 0–100, 101–200, 201–400, >400 days after HSCT in BM, Fig. 6j). In addition, we observed that these PD-1^+^CD8^+^ T_SCM_-like regulatory T cells were homing to recipient bone marrow and it is well established that the homing of HSCs to the bone marrow niches was essential to a successful engraftment post-transplantation.^68^ PD-1^+^CD8^+^ T_SCM_-like regulatory cells in recipients’ PB eventually decreased to a level similar to those of healthy donors after 4–8 months post allo-HSCT (4.493 ± 0.3237, Fig. 6i). These results indicated that the higher level of these cells plays dual functions, especially in the susceptible period of aGVHD and infection.^9^ The mean of PD-1^+^CD8^+^ T_SCM_-like regulatory cell counts was also calculated, with results similar to the change in proportion (PB: 4986, 2023, 247.3/mL respectively 0–2, 2–4, 4–8 months after HSCT; BM: 8073, 861.7, 136.7, 225.7/mL respectively 0–100, 101–200, 201–400, > 400 days after HSCT).

### G-CSF induces PD-1^+^CD8^+^ T_SCM_-like regulatory T cells by BCL6

**Fig. 7:**
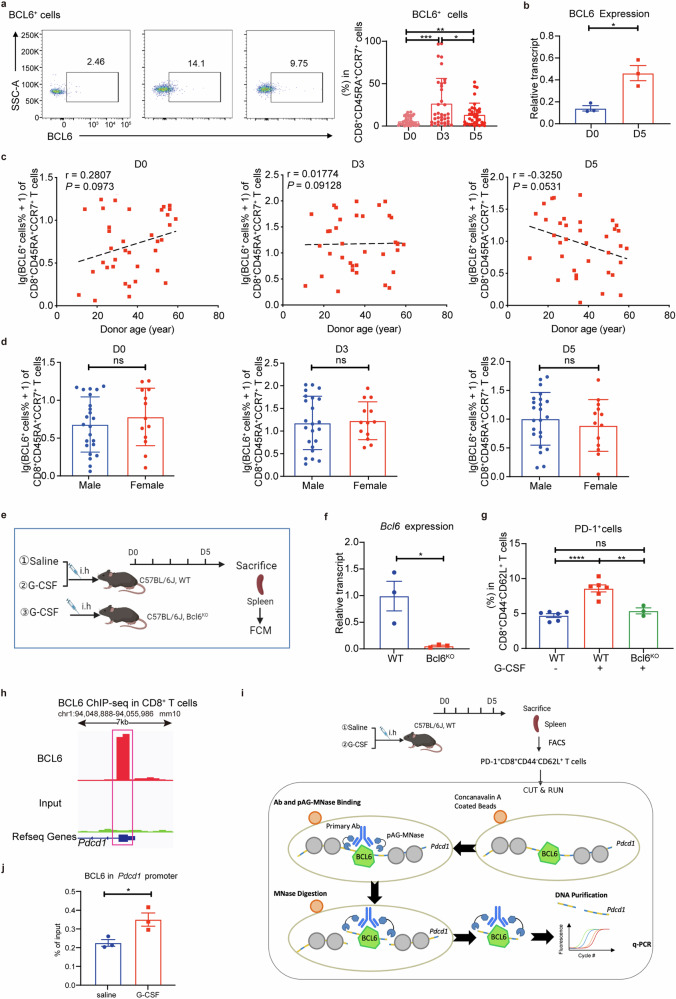
**G-CSF induces the increase of PD-1**^**+**^**CD8**^**+**^ **T**_**SCM**_**-like regulatory T cells via BCL6 in both mouse in vivo model and clinical donors. a**–**d** G-CSF mobilization increased BCL6 expression in CD8^+^CD45RA^+^CCR7^+^ T cells in donor PB and the expression was not correlated with donor age and gender before or after mobilization. **a** Representative plots and the percentages of BCL6^+^ cells in the PB of healthy donors before and after G-CSF mobilization (*n* = 36). **b** BCL6 expression in CD8^+^CD45RA^+^CCR7^+^ T cells in the PB of healthy donors before (D0, blue) and after G-CSF (D5, red) mobilization by q-PCR (*n* = 2–3). **c** Lack of correlation between the normalized frequencies of BCL6^+^ cells and the donor age before and after G-CSF mobilization (D0, D3, D5, *n* = 36). **d** Lack of correlation between the normalized frequencies of BCL6^+^ cells in CD8^+^CD45RA^+^CCR7^+^ T cells and the donor gender before and after G-CSF mobilization (D0, D3, D5) (*n* = 36). **e**–**g** G-CSF mobilization in the mouse induces the increase of PD-1^+^CD8^+^ T_SCM_-like regulatory T cells by BCL6 in vivo. **e** Outline of injecting rhG-CSF subcutaneously (i.h) to C57BL/6J mice (WT or Bcl6^KO^) for 5 days, mimicking clinical mobilization in healthy donors: (1) saline-treated C57BL/6J WT mice; (2) G-CSF-treated C57BL/6J WT mice; (3) G-CSF-treated C57BL/6J Bcl6^KO^ mice. The image was created using Biorender (https://biorender.com/). **f** *Bcl6* gene expression in CD8^+^CD44^−^CD62L^+^ T cells in the spleen of Bcl6^fl/fl^ Cre^ERT2^ mice before (WT, blue) and after Tamoxifen (TAM) (Bcl6^KO^, red) treatment by q-PCR (*n* = 3). **g** The percentage of PD-1^+^ cells in CD8^+^CD44^−^CD62L^+^ T cells in the spleen of saline-treated WT mice (blue), G-CSF-treated WT mice (red) and G-CSF-treated Bcl6^KO^ mice (green) by FCM (*n* = 6). **h** Visualization of BCL6 binding to the *Pdcd1* gene in gse182034 with The Integrative Genomics Viewer (IGV). **i** Outline shows the workflow of the CUT & RUN q-PCR using the G-CSF mobilization mouse model. The image was created using Biorender (https://biorender.com/). **j** The deposition of BCL6 at the *Pdcd1* gene in splenic PD-1^+^CD8^+^CD44^−^CD62L^+^ T cells from saline-treated (blue) and G-CSF-treated (red) WT mice (*n* = 3). Data are presented as the mean ± SEM, *P < 0.05, **P < 0.01, ***P < 0.001, ****P < 0.0001

We observed a significant increase of BCL6 expression scRNA-seq (Fig. 2g) and given that BCL6 promotes PD-1 expression in Tfh cells,^45,46^ we speculated that G-CSF might promote PD-1 expression in CD8^+^CD45RA^+^CCR7^+^ cells through BCL6. To this end, we first measured the BCL6 expression in the CD8^+^CD45RA^+^CCR7^+^ cells in PB of clinical donor. BCL6 showed a significant increase on D3 of G-CSF mobilization and a gradual decrease in D5 (Fig. 7a), which indicated that G-CSF induced BCL6 expression prior to PD-1. *BCL6* mRNA level also increased significantly (Fig. 7b). Similar to PD-1, BCL6 expression was not correlated with donor age or gender before or after mobilization (Fig. 7c, d). However, the cut-off value of donor BCL6^+^CD8^+^CD45RA^+^CCR7^+^ T cells could not be determined, suggesting that this subset was not associated with aGVHD. And PD-1, rather than BCL6, was an indicator for recipient prognosis.

To study how BCL6 regulates PD-1 expression, we constructed lentiviral vectors to overexpress or knock down BCL6 in human CD8^+^CD45RA^+^CCR7^+^ T cells in vitro (Supplementary Fig. 12a, c). BCL6-overexpression CD8^+^CD45RA^+^CCR7^+^ T cells expressed higher PD-1 and exhibited the PD-1^+^CD8^+^ T_SCM_-like regulatory T cell phenotype (Supplementary Fig. 12a, b). BCL6-knockdown CD8^+^CD45RA^+^CCR7^+^ T cells expressed lower PD-1 and exhibited a phenotype different from PD-1^+^CD8^+^ T_SCM_-like regulatory T cells (Supplementary Fig. 12c, d). These results indicate that BCL6 regulates PD-1 expression in CD8^+^CD45RA^+^CCR7^+^ T cells.

Previous studies have shown that G-CSF plays a role through G-CSFR and downstream STAT3 phosphorylation.^36,69^ Our scRNA-seq results also showed G-CSFR and STAT3 upregulation after G-CSF mobilization. Therefore, we measured the expressions of G-CSFR, p-STAT3, BCL6, and PD-1 in CD8^+^CD45RA^+^CCR7^+^ T cells of healthy individuals with G-CSF treatment in vitro. G-CSF treatment significantly increased G-CSFR and p-STAT3 expressions (Supplementary Fig. 13a). BCL6 and PD-1 showed similar increases (Supplementary Fig. 13a) with mRNA levels also upregulated (Supplementary Fig. 13b). These results suggested that G-CSF induced the expressions of BCL6 then PD-1 in CD8^+^CD45RA^+^CCR7^+^ T cells through G-CSFR and p-STAT3.

We further confirmed that BCL6 regulated PD-1 expression in mice by constructing Bcl6^KO^ mice (Fig. 7e, f). We found that PD-1 expression in splenic CD8^+^CD44^−^CD62L^+^ cells of G-CSF-treated Bcl6^KO^ mice was the same compared to that of saline-treated WT mice. Their PD-1 expressions were significantly lower than that in the G-CSF-treated WT group (Fig. 7g). Therefore, BCL6 is critical for G-CSF-induced PD-1 expression in vivo, and we conclude that G-CSF induces PD-1^+^CD8^+^ T_SCM_-like regulatory T cell expansion via BCL6.

Since our results show that this PD-1^+^CD8^+^ T_SCM_-like regulatory T cell is modulated by transcription factor BCL6, we next examined whether BCL6 directly regulates PD-1(*Pdcd1*) transcription in PD-1^+^CD8^+^ T_SCM_-like regulatory T cell. To this end, we re-analyzed a previously published chromatin immunoprecipitation sequencing (ChIP-seq) data set of mice CD8^+^ T cells,^48^ and found that BCL6 bound to the *Pdcd1* promoter region (Fig. 7h). We also verified this interaction in PD-1^+^CD8^+^ T_SCM_-like regulatory T cells in G-CSF mobilization mouse model (Fig. 7i). The Cleavage Under Targets & Release Using Nuclease (CUT&RUN) analysis of splenic PD-1^+^CD8^+^CD44^−^CD62L^+^ T cells in mice revealed that the binding of BCL6 at the *PDCD1* promoter increased upon G-CSF-treatment (Fig. 7j). Therefore, we speculate that the underlying mechanism by which G-CSF stimulates the BCL6 expression may due to the direct recruitment of BCL6 to PD-1 gene promoter region.

## Discussion

Allo-HSCT has been widely used to treat malignant hematologic diseases. A key step to ensure successful allo-HSCT is to find donors who provide T cells that balance the GVHD and GVL in recipients which not only facilitates immune reconstitution but also inhibits relapse. Current gold standard for clinical selection of donors is HLA compatibility with consideration of other factors including donor-specific antibodies, ABO matching, donor age and gender.^17^ However, due to the highly polymorphic features at the HLA loci, only 30% of recipients will find an HLA-matched donor.^70^ As a consequence, haploidentical donor selection is being increasingly used in HSCT because of the expanded donor pool.^71^ Nonetheless, the current donor selection standard for allo-HSCT which prefers younger male is still mainly based on clinical correlation research and lacks molecular and cellular markers that are causally to favorable prognosis in recipients.^19,20^ Recent efforts to identify donor unique cell populations that balance the GVHD/GVL are inconclusive. On one hand, clinical data showed that donor CD45RA^+^ T cells were associated with higher GVHD incidence,^72–74^ on the other, cord blood CD45RA^+^ T cells correlated with reduced GVHD and higher GVL in recipients.^75–77^ These contradicted findings may be explained by the scRNA-seq results which revealed that the CD45RA^+^ T cells are heterogenous.^78^ Therefore, the identification and mechanistic study of different subsets of CD45RA^+^ T cells are necessary to elucidate their distinct roles, including alleviating GVHD, promoting GVL, anti-infection and reconstitution in recipients after transplantation, which may facilitate the development of novel clinical allo-HSCT strategies. There are a few molecules shown to regulate the balance between GVHD and GVL and affect the outcome of allo-HSCT recipients. Meanwhile, most studies focus on biomarkers in recipients for predicting GVHD. Recently, PD-1 and its ligand PD-L1 are reported to alleviate GVHD in preclinical studies in mice possibly through suppression of Th1 and CD8^+^ T cells.^40–42^ In addition, G-CSF primed donor graft alleviates GVHD in some clinical recipients in previous research,^36–38^ and our recent mechanism study demonstrated that G-CSF directly restrained T cell activation through G-CSFR.^30^ Consistently, we also revealed here that G-CSF induced G-CSFR expression in CD8^+^CD45RA^+^CCR7^+^ T cells of donor peripheral blood.

In this study, we aimed to identify donor CD4^+^ and CD8^+^ T cell subsets that attenuate GVHD while promoting GVL for the development of optimal donor selection indicators and therapeutic targets for GVHD treatment. Intriguingly, we found that PD-1^+^CD8^+^CD45RA^+^CCR7^+^ T cells were significantly expanded in the peripheral blood of G-CSF-mobilized donors. More importantly, this subset was positively correlated with alleviated aGVHD in recipients which showed a ~2-fold lower infection rate and overall death. Interestingly, PD1^+^CD8^+^CD45RA^+^CCR7^+^ T cells exhibited features of both CD8^+^ Treg and CD8^+^ Teff and expressed T_SCM_ makers, including CXCR3, CD95 and CD69 as well as CD49d, CD107a, perforin, granzyme B, IFN-γ and IL-2, and CD103, CD73 which are also expressed in CD8^+^ Treg that alleviate GVHD.^59,79,80^ We further confirmed these findings in clinical donors by functional experiments in vitro, which demonstrated this donor-derived T cell subset mobilized by G-CSF had a dual function of immune regulation and cytotoxicity.

T_SCM_ is a population of rare but long-lived T cell, which can rapidly differentiate into effector memory T cells (T_EM_) or central memory T cells (T_CM_), while maintaining their precursor potential.^50^ We find in this study that *ZNF683* is highly expressed in PD1^+^CD8^+^CD45RA^+^CCR7^+^ T cells of G-CSF mobilized donor PB. In line with our results, recent studies have shown that *ZNF683* not only marks alloreactive and cytotoxic CD8^+^ T cells but also is critical for maintaining the persistence and quiescence of CD8^+^ T cells in allo-HSCT recipients.^51,81^ We speculate that *ZNF683* may be related to the dual functions of PD-1^+^CD8^+^CD45RA^+^CCR7^+^ T cells and its mechanism and function remains to be explored. Altogether, PD-1^+^CD8^+^CD45RA^+^CCR7^+^ T cells have unique T_SCM_ expression markers and possess features of both regulatory and effector T cells, and they correlate with ameliorated aGVHD and anti-infection in post allo-HSCT patients. Therefore, we define this novel T cell subset as PD-1^+^CD8^+^ T_SCM_-like regulatory cells. We further confirmed the existence of the equivalence of this subset in mice (PD-1^+^CD8^+^CD44^−^CD62L^+^ T cells). Markedly, this subset also expanded in G-CSF-treated donor mice and caused ameliorated aGVHD in recipient mice, like human PD-1^+^CD8^+^ T_SCM_-like regulatory cells.

PD-1/PD-L1 blockade has been shown to enhance aGVHD and cGVHD in mouse models.^40,42^ Interestingly, our study also revealed that in PD-1 knockout mice, donor CD8^+^CD44^−^CD62L^+^ cells lost their immune regulatory activities even after G-CSF mobilization. This finding not only confirmed the causal effect of PD-1/PD-L1 on T cell activities in aGVHD but also underscored the importance of G-CSF treatment in PD-1^+^CD8^+^ T_SCM_-like regulatory cells induction to alleviate aGVHD. Although we previously found that G-CSF directly restrained T cell activation in G-BM,^30^ how it affects different subsets of lymphocytes in different donors’ peripheral blood remains elusive. G-CSF also elevates the expression of PD-1 in CD4^+^ T cells^82–84^ and regulates BCL6 in Tregs.^85^ Interestingly, BCL6 has been shown to induce PD-1 expression in mice CD4^+^ Tfh cells.^45,46^ Previous reports revealed that STAT3 regulates BCL6 in T cells^86,87^ and consistently, we confirmed that G-CSF induces BCL6 expression through STAT3 by in vitro experiments. Altogether, our study first reveals that the transcription factor BCL6 specifically drives the differentiation of PD-1^+^CD8^+^ T_SCM_-like regulatory cells in donor G-PBSC, which may serve as an important cellular immune mechanism for the balance of aGVHD, anti-infection and GVL activities in recipients. Given that the same G-CSF mobilization may cause different expansion of PD-1^+^CD8^+^ T_SCM_-like regulatory cells in individual donors, the abundance of this subset may serve as a potential clinical indicator in allo-HSCT for haploidentical donor selection. More importantly, due to the dual Treg and Teff functions of this subset, it may be a new type of cell therapy for not only allo-HSCT but also auto-immune diseases. In addition, PD-1^+^CD8^+^ T_SCM_-like regulatory cells can survive for a long time in recipients to resist early risk events such as the suppression of aGVHD and infection, which may help to promote immune reconstitution.^88,89^ The underlying mechanism of the PD-1^+^CD8^+^ T_SCM_-like regulatory subset, including the establishment of bone marrow niche, the relevant biological pathways, the interaction of these T cells with the bone marrow microenvironment and deep function such as long-term anti-infection in recipients, are tasks waiting for us to explore. In this study, we demonstrated that in the clinical recipients, the donor-derived PD-1^+^CD8^+^ T_SCM_-like regulatory cells were able to home to recipient bone marrow and existed for a long period, which may promote successful engraftment and immune reconstitution. In future studies, we will focus on the underlying mechanism of the PD-1^+^CD8^+^ T_SCM_-like regulatory subset, including the establishment of bone marrow niche, the relevant biological pathways, the interaction of these T cells with the bone marrow microenvironment and its regulation on long-term anti-infection in recipients. Nevertheless, the dual Treg and Teff activities of PD-1^+^CD8^+^ T_SCM_-like regulatory T subset make it a promising cell therapy candidate for aGVHD post allo-HSCT.

In summary, we have identified the PD-1^+^CD8^+^ T_SCM_-like regulatory cells possessing unique immunological properties of both alleviating aGVHD and preserving GVL, which have the potential to help optimize current allo-HSCT donor selection and to facilitate development of a new T cell therapy for GVHD in allo-HSCT in addition to recently FDA-approved MSC clinical products and preclinical MDSC immunotherapy,^90,91^ as well as auto-immune diseases.

## Materials and methods

### Human samples

Donor selection, HLA-typing, conditioning therapy, and GVHD prophylaxis have been previously described1.^92–95^ Briefly, donors were mobilized with recombinant human G-CSF (rhG-CSF) (Filgrastim, Kirin, Japan) at 5 μg/kg daily injected subcutaneously (i.h) for 5 consecutive days before transplantation. The transplanted grafts were all G-PBSC only without bone marrow from either matched sibling donors or haploidentical donors. The donors’ and recipients’ characteristics are described in Supplementary Table 1. aGVHD was diagnosed based on the Glucksberg-Seattle criteria and gastrointestinal (GI) aGVHD was confirmed by colonoscopy and biopsy.^96^ The complete response (CR) to aGVHD treatment was defined according to the published criteria.^97^ Peripheral blood samples from donors were continuously collected and monitored before and after 3 and 5 days for markers analysis by FCM and q-PCR. Peripheral blood and bone marrow samples from recipients were collected and monitored after transplantation by FCM.

### Mice

8- to 12-week-old female C57BL/6J (H-2kb, CD45.2^+^) and BALB/c (H-2kd, CD45.2^+^) mice (SLAC, Shanghai, China) were bred and maintained in the specific-pathogen-free animal facility at the Department of Laboratory of Animal Science, Shanghai Jiao Tong University School of Medicine. PD-1-deficient (PD-1^−/−^) mice (in C57BL/6J background) were kindly provided by Dr. Yong Yu (Tongji University). Bcl6^fl/fl^ Cre^ERT2^ mice (in C57BL/6J background) were kindly provided by Dr. Chuanxin Huang (Shanghai Jiao Tong University School of Medicine) and treated for a period of 5 days by oral gavage (PO) with 0.1 mL (2 mg) Tamoxifen (TAM) to knock out *Bcl6* and analyzed by q-PCR.

### Flow cytometry

Fluorochrome-conjugated monoclonal antibodies (Supplementary Table 4) and their isotype controls were purchased from BioLegend, BD Biosciences, or eBioscience. All reagents were titrated before use to determine optimal concentrations. Surface markers were measured by incubating cells for 20 min at room temperature. For intracellular cytokines analysis, cells were cultured in RPMI-1640 medium containing 10% FBS and stimulated with a cell stimulation cocktail (plus protein transport inhibitors) (eBioscience) for 4 h. Cells were then harvested for surface staining and permeabilized using a Cytofix/Cytoperm Kit (BD Biosciences), according to the manufacturer’s instructions. Intracellular molecules were then incubated for 30 min at room temperature. Dead cells were eliminated from the analysis using Fixable Viability Dye (eBioscience). For the comprehensive multicolor analysis, Comp Beads (BD Biosciences) and single-color staining were used for the adjustment of compensation. Data were acquired using an LSRFortessa (BD Biosciences) and analyzed with FlowJo software version 10.

### Single-cell RNA sequencing

Donor peripheral CD8^+^CD45RA^+^CCR7^+^ T cells were sorted from PBMCs with Human Naïve CD8^+^T Cell Isolation Kit (Miltenyi). In the first step, non-T_N_, including memory/effector T cells, activated T cells, and NK cells are depleted using biotin-conjugated antibodies against CD45RO, CD56, CD57, and CD244. In the second step, naïve CD8^+^ T cells are isolated by positive selection using CD8 MicroBeads and collected after resuspension with PBS on ice to make sure the cell viability is >85%. According to the Singleron GEXSCOPER operation steps, the single-cell suspension with a concentration of 1 × 10^5^ cells/mL was loaded onto the GEXSCOPER microfluidic chip. The magnetic bead paired with a cell in each microwell had a unique cell label and multiple molecular labels for capturing RNA and then collected for constructing the single-cell RNA-seq library according to the manufacturer’s protocols (Singleron GEXSCOPE Single Cell RNA-seq Library Kit, Singleron Biotechnologies). The library was sequenced on the Illumina HiSeq X using 150 bp paired-end reads. R package Seurat (version 4.3.0.1)^98^ was used to explore QC metrics and filter cells: 200 < gene counts, < 4500 per cell, and the percentage of mitochondrial genes < 12%. After gene expression normalization and principal component analysis, filtered count matrices were combined with the Harmony package (version 1.0).^99^ Dimension reduction was then performed using Harmony-corrected principal components and visualized cells with the UMAP algorithm. The first 20 principal components were used to generate an initial clustering using the Seurat community detection algorithm to identify cell clusters and non-CD8^+^ T cells were removed from the matrices. Differentially expressed genes between different groups or subsets were identified by the Seurat function “FindAllmarkers” with the following parameters: min. pct = 0.05, return.thresh = 0.01, logfc.threshold = 0.25. *P*-values of genes distinctly expressed between samples or subsets were performed by a nonparametric Wilcoxon rank-sum test, and adjusted *P*-values (*P*adj) were based on Bonferroni correction. The control features were selected from the feature genes of each subset. Feature plots, dot plots, and heatmaps were also generated using “Seurat” and “pheatmap” implemented functions. All gene expressions with significant differences were tested by a nonparametric Wilcoxon rank-sum test (*P* < 0.05). The gene ontology (GO) analysis and Gene set enrichment analysis (GSEA) were performed by the R package ClusterProfiler (version 4.8.3).^100^ GO terms were calculated with the following parameters: ont = “ALL”, pAdjustMethod = “BH”, pvalueCutoff = 0.05, qvalueCutoff = 0.2. Statistical analysis was performed by hypergeometric distribution. GSEA terms were performed with following parameters: exponent = 1, minGSSize = 10, maxGSSize = 500, pvalueCutoff = 0.05, pAdjustMethod = “BH”.

### In vitro functions of human peripheral PD-1^+^CD8^+^CD45RA^+^CCR7^+^ cells

Sorted PD-1^+^CD8^+^CD45RA^+^CCR7^+^ and PD-1^−^CD8^+^CD45RA^+^CCR7^+^ cells of clinical donors on D5 were effector cells in two functional experiments. For the proliferation assay, T cells of healthy donors were stained by CellTrace Violet Cell Proliferation Kit (CTV, Invitrogen) for 20 min at 37 °C and then seeded in 96-well round-bottom plates plus anti-CD3/CD28 antibodies (eBiosciences). Effector cells were added at 1:1 and 2:1 effector-to-target (E:T) ratios. After co-culturing for 3 days, CTV negative T cells represent proliferating T cells were analyzed by flow cytometry. Proliferation rate = proliferating T cells/ total T cells. Inhibition rate = (Proliferation rate of T cells cocultured without effector cells − Proliferation rate of T cells cocultured with effector cells)/Proliferation rate of T cells cocultured without effector cells. For the cytotoxicity assay, Raji-GFP cells were seeded in 96-well round-bottom plates at a concentration of 1 × 10^4^ cells/well. Effector cells were added at 1:1 and 10:1 effector-to-target (E:T) ratios. After co-culturing for 4 h, dead rate of Raji-GFP cells was measured with Fixable Viability Dye (FVD, Invitrogen) by flow cytometry. Cytotoxicity rate = dead rate of Raji-GFP cells cocultured with effector cells − dead rate of Raji-GFP cells cocultured without effector cells. In our study, cells were all cultured in RPMI 1640 containing 10% FBS and incubated at 37 °C/5% CO_2._

### In vivo effects of rhG-CSF on T cells in mice

WT, PD-1^−/−^ and Bcl6^KO^ mice (C57BL/6J) were injected subcutaneously (i.h) with rhG-CSF diluted with saline at a dose of 125 μg/kg twice daily for 5 days. Mice were sacrificed on day 5 after rhG-CSF mobilization, and PD-1 expression in CD8^+^CD44^−^CD62L^+^T cells in the spleen was analyzed by FCM.

### aGVHD and GVL model

BALB/c mice were lethally irradiated at 8 Gy using RS 2000 pro X-ray biological Irradiator (Rad Source Technologies, Inc., USA) as a split dose with a 4-h interval on D-1. On D0, each BALB/c recipient mouse was injected intravenously (i.v) with 5 × 10^6 ^T cell-depleted bone marrow (TCD-BM) sorted from WT C57BL/6J donor and 5 × 10^5^ rhG-CSF or saline-treated splenic CD8^+^CD44^−^CD62L^+^ T cells from WT or PD-1^−^^/^^−^ C57BL/6J mice. TCD-BM and splenic CD8^+^CD44^−^CD62L^+^ T cells were purified respectively with anti-CD90.2 MicroBeads and CD8^+^ naïve T cells isolation kit (Miltenyi Biotec), according to the manufacturer’s protocols. Based on aGVHD experiments, GFP-A20 cells were used in GVL experiments. Briefly, recipients received grafts with 5 × 10^5^ A20 cells and were monitored every 4 days to assess survival and weight change after transplantation. A20 cells in peripheral blood after transplantation were analyzed with FCM.

### Evaluation of aGVHD and histopathological analysis

After transplantation, mice were weighed every 2 days, and the degree of systemic aGVHD was assessed by a clinical scoring system including five clinical parameters: weight loss, posture, activity, fur ruffling, and skin integrity, as published previously.^10^ For histopathological analysis, tissues from GVHD target organs (liver, lung, small intestine, colon, skin) and spleen specimens of recipients were fixed with 4% paraformaldehyde, embedded in paraffin, sectioned, mounted on slides, and stained with hematoxylin and eosin. Two slides/organs were evaluated and scored by a pathologist blinded to the experimental group using a scoring system described previously.^101,102^

### Immunofluorescence

Liver, colon, and small intestine of recipient mice were obtained for immunofluorescence when GVHD onset. 4-μm-thick formalin-fixed, paraffin-embedded whole tissue sections were stained with primary antibodies, sequentially with NEON-TSA kit (Yuanxibio, Shanghai, China) and finally with DAPI. Specifically, deparaffinized slides were incubated with rabbit anti-mouse CD8 antibody (CST) for 1 h at room temperature and then treated with goat anti-rabbit horseradish peroxidase (HRP)-conjugated secondary antibody for 10 min. Slides were then labeled with NEON-TSA 570 for 10 min and washed in TBST buffer. Slides were transferred to preheated citrate solution (90 °C), heated for 15 min using a microwave set at 20% of maximum power, and cooled in the same solution to room temperature. The same process was repeated for rabbit anti-mouse PD-1 antibody (CST)/NEON-TSA 520. 4’,6-diamidino-2-phenylindole (DAPI) was used to stain the nucleus, and images were acquired with a Leica TCS SP8 confocal microscope (Leica Microsystems, Wetzlar, Germany).

### Virus preparation and cell transduction

hBCL6 CRISPR/Cas9 knockout lentiviral vector was purchased from Ubigene. hBCL6 overexpression lentiviral vector was generated as follows: BCL6 cDNA fragment was obtained by removing the BCL6 gene sequence from the MSCV-BCL6-IRES-GFP plasmid (Addgene, Plasmid 31391) and was ligated into the BamHI and XhoI site of pLVX-3Flag-MCS-Puro plasmid to make pLVX-3Flag-BCL6-MCS-Puro plasmid. Lentiviruses above were produced using the second-generation production system. HEK293T cells were maintained in 10% FBS-containing Dulbecco’s modified Eagle medium. Cells at 70% confluence were cotransfected with PAX, VSVG, and lentiviral vectors expressing the construct of interest or control lentiviral vectors. Medium was replenished 12 h after transfection, and the supernatant was collected after 48 h and was passed through a 0.45-μm filter, pooled, and used either fresh or snap-frozen. For cell transduction, sorted CD8^+^CD45RA^+^CCR7^+^ T cells from human PBMCs were stimulated for 24 h in 6-well plates with anti-CD3/CD28 antibodies. Then cells were incubated with viral supernatants in the presence of polybrene (Yeasen), centrifuged for 1 h at 1200 rpm at 25 °C, and incubated overnight. Following 2 additional days of culturing in the medium with IL-7 (Peprotech), infected cells were selected with puromycin (Yeasen).

### In vitro effects of rhG-CSF on human peripheral T cells

Sorted CD8^+^CD45RA^+^CCR7^+^ T cells from human PBMCs were cultured in 96-well plates supplemented with anti-CD3/CD28 antibodies and added with 0 or 500 ng/mL rhG-CSF (Filgrastim, Kirin, Japan) and cultured for 48 h. After culture, cells were harvested, counted, and subsequently evaluated G-CSFR, p-STAT3, BCL6, and PD-1 expression by FCM and *CSF3R*, *STAT3*, *BCL6*, and *PDCD1* gene expression by q-PCR.

### Quantitative real-time PCR analysis

Total RNA was isolated from sorted CD8^+^CD45RA^+^CCR7^+^ cells of human PBMCs and sorted CD8^+^CD44^−^CD62L^+^ cells of spleen in mice. cDNA was synthesized with a cDNA reverse transcription kit (Yeason). q-PCR assays were performed with One Step RT-qPCR Probe Kit (Yeason) on ViiA 7 Real-Time PCR System (Applied Biosystems). All data were normalized on the basis of mRNA for β-actin, and gene relative copy numbers were calculated. The primers for q-PCR are listed below in Supplementary Table 4.

### Cleavage under targets and release using nuclease (CUT&RUN)

CUT&RUN experiment was performed by CUT&RUN Assay Kit (CST), including PD-1^+^CD8^+^CD44^−^CD62L^+^ T cell preparation (isolated from spleens of saline-treated and G-CSF-treated mice), binding of Concanavalin A beads and primary antibody, binding of pAG-MNase, enzyme DNA digestion and diffusion, preparation of the input sample, DNA purification and quantification of DNA by qPCR. Primers for q-PCR listed in Supplementary Table 4 were designed by IGV and Primer3plus.

### Statistics

Paired and unpaired samples for two groups were compared using the Wilcoxon’s test and nonparametric Mann–Whitney U test respectively. Survival comparisons were performed using the log-rank test. For normally distributed values, a Pearson correlation analysis was performed. *P*-values < 0.05 were considered statistically significant. Data were processed in GraphPad Prism 10.0 software.

## Supplementary information

Supplementary Materials

Supplementary files of GO and GSEA analysis

## Data Availability

The scRNA-seq data presented in this paper have been deposited in the Genome Sequence Archive in the National Genomics Data Center, Chinese Academy of Sciences (https://ngdc.cncb.ac.cn/gsa‐human/), reference number: HRA010395 or BioProject number: PRJCA035662. All data employed in this study can be obtained upon a reasonable request to the corresponding author.
