# Supplementary material for: A donor PD-1+CD8+ TSCM-like regulatory subset mobilized by G-CSF alleviates recipient acute graft-versus-host-disease
Source: Signal Transduct Target Ther. 2025 Apr 2;10:120. doi: 10.1038/s41392-025-02183-1 (PMC11965471; doi:10.1038/s41392-025-02183-1)
Supplement: Supplementary file 1 — Supplementary Materials [file 41392_2025_2183_MOESM1_ESM.docx]

Supplementary Materials for

A donor PD-1^+^CD8^+^ T_SCM_-like regulatory subset mobilized by G-CSF alleviates recipient aGVHD

Dan Liu^1,2#^, Xue Wang^1#^, Yuheng Han^1^, Jing Wang^3^, Yidan Sun^3^, Yafei Hou^1^, Qian Wu^1^, Cong Zeng^1^, Xuping Ding^1^, Yingjun Chang^2^, Jiong Hu^3*^, Xiaojun Huang^2,3*^, Liming Lu^1*^

Correspondence to:[youlanda2009@sjtu.edu.cn](mailto:youlanda2009@sjtu.edu.cn)

**This PDF file includes:**

Figures S1 to S13

Tables S1 to S4

Captions for Data S1 to S9

**Other Supplementary Materials for this manuscript include the following:**

Data S1 to S9


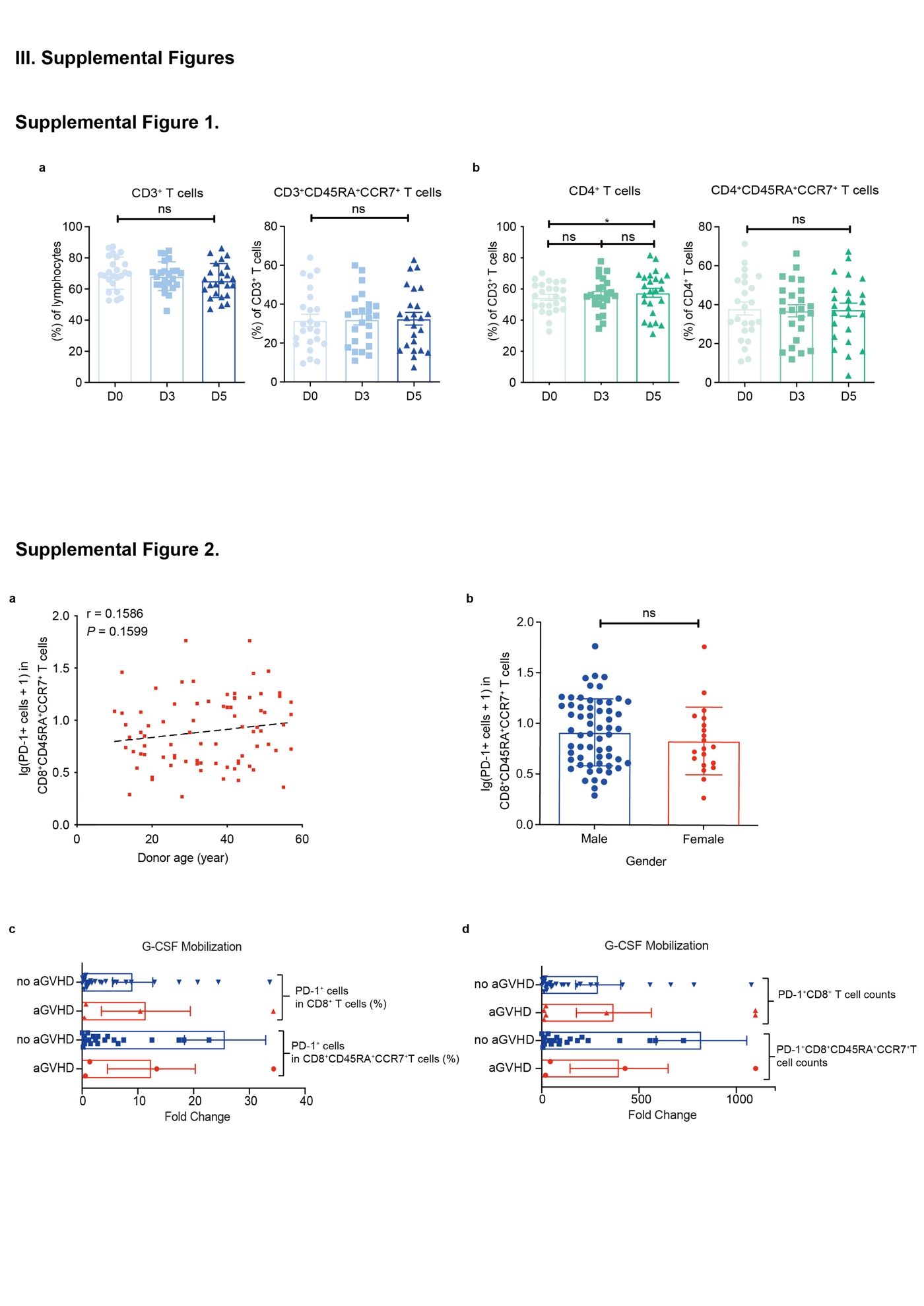


Figure. S1.

**Frequencies of CD3^+^ and CD4^+^ T cell and their naïve subsets (CD45RA^+^CCR7^+^) in donors during G-CSF mobilization.**

**a** The percentages of CD3^+^ T cells in total lymphocytes and CD45RA^+^CCR7^+^ T cells in CD3^+^ T cells before (D0) and after three (D3) or five (D5) days of G-CSF mobilization to the healthy donors by FCM (*n*=24). **b** The percentages of CD4^+^ T cells in total lymphocytes and CD45RA+CCR7^+^ T cells in CD4^+^ T cells before (D0) and after three (D3) or five (D5) days of G-CSF mobilization to the healthy donors by FCM (*n* =24). Data are presented as the mean ± SEM, **P* < 0.05.


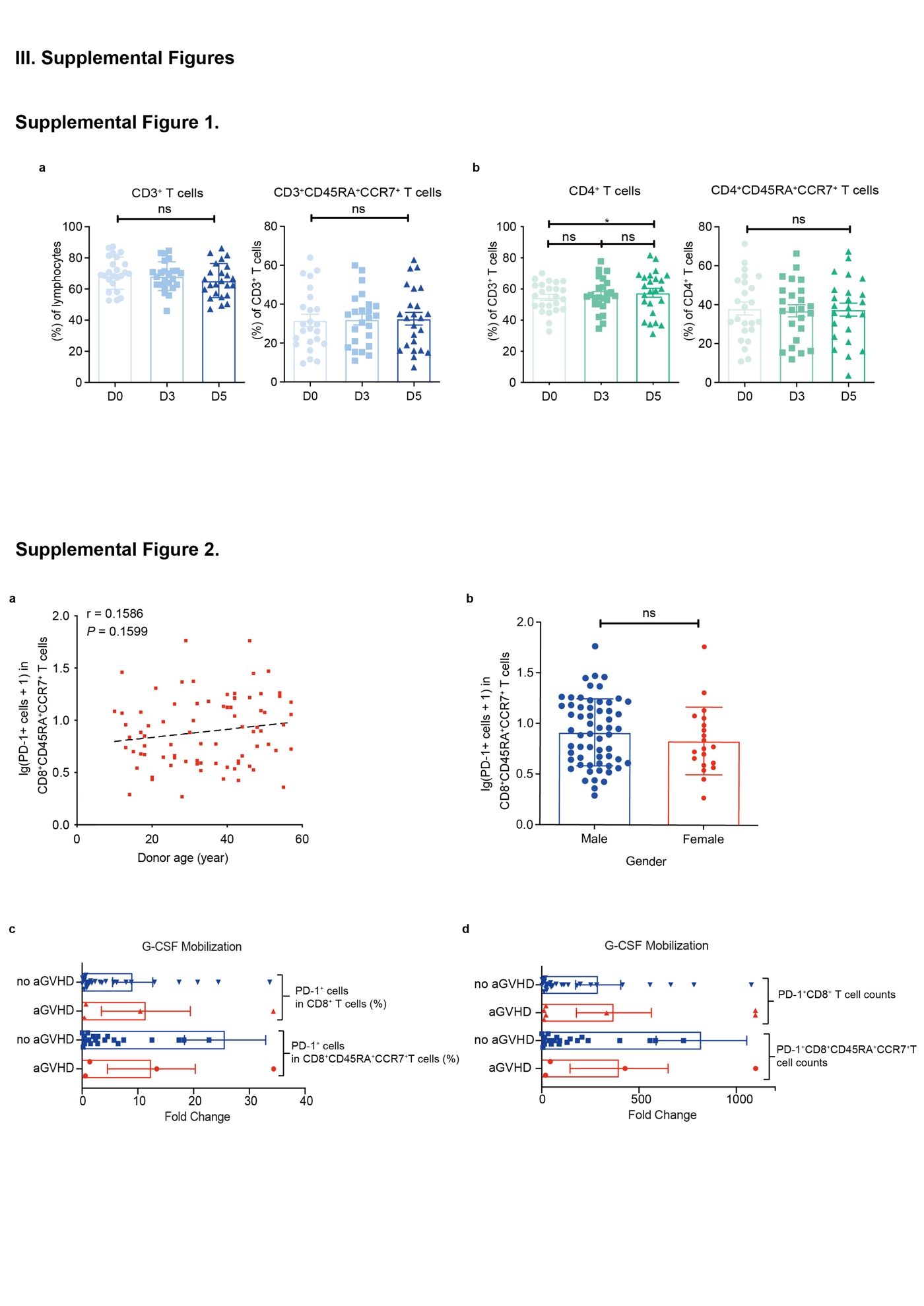


Figure. S2.

**The expression of PD-1^+^ cells was analyzed in donors of different ages and genders, and the fold change was measured after G-CSF mobilization in groups based on recipients’ outcomes of aGVHD.**

**a** Lack of correlation between the normalized frequencies of PD-1^+^ cells in CD8^+^CD45RA^+^CCR7^+^ T cells on D5 and the donor age by FCM (*n*=80). **b** Lack of correlation between the normalized frequencies of PD-1^+^ cells in CD8^+^CD45RA^+^CCR7^+^ T cells on D5 and the donor gender by FCM (*n*=80). **c** The increase of PD-1^+^ cells in CD8^+^ T cells (%) and PD-1^+^ cells in CD8^+^CD45RA^+^CCR7^+^ T cells (%) in donor PB corresponding to the recipients with (red) and without (blue) aGVHD after G-CSF mobilization by FCM (*n*=33). **d** The increase of PD-1^+^CD8^+^ T cell counts and PD-1^+^CD8^+^CD45RA^+^CCR7^+^ T cell counts in donor PB corresponding to the recipients with (red) and without (blue) aGVHD after G-CSF mobilization by FCM (*n*=33). Data are presented as the mean ± SEM.


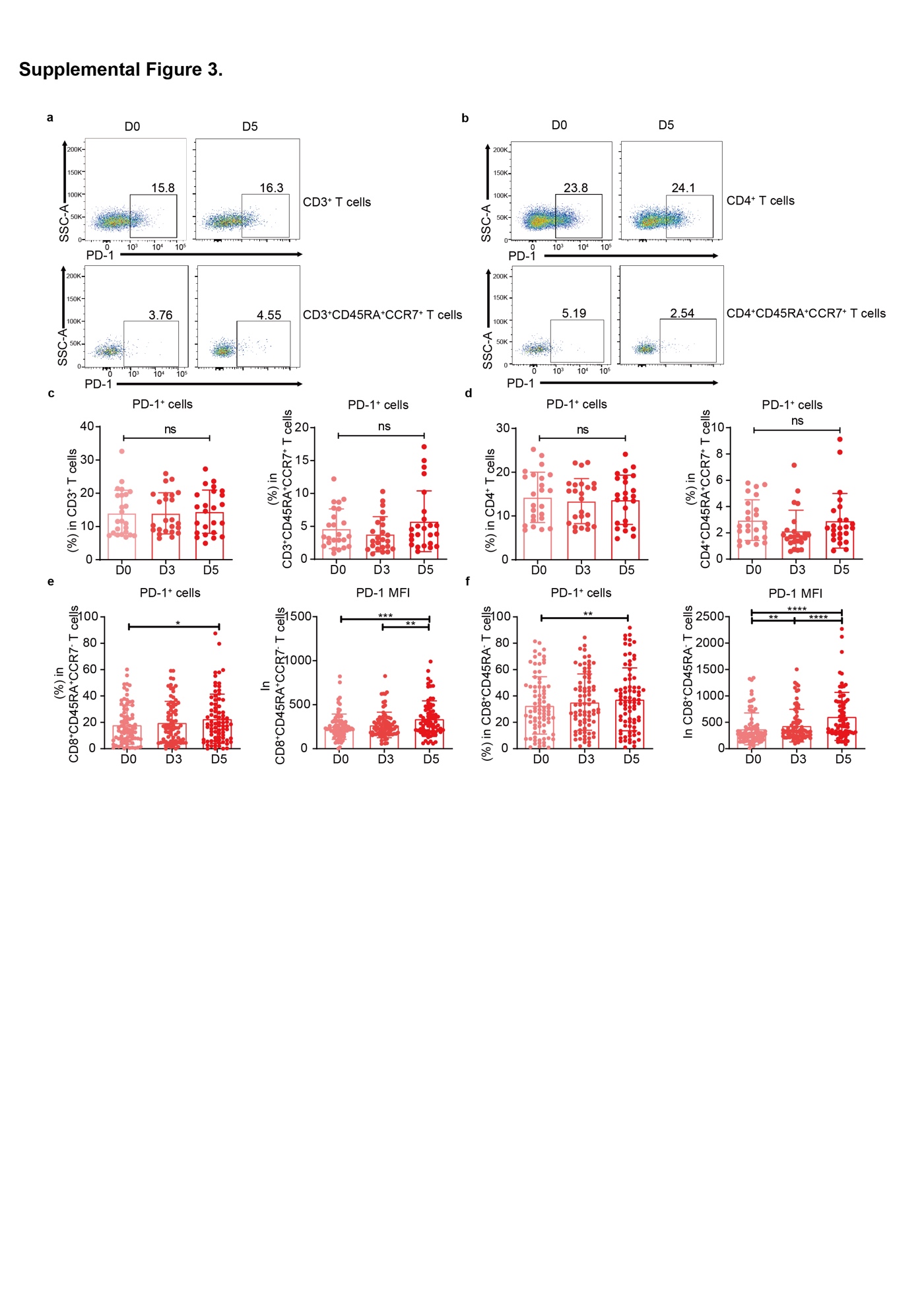


Figure. S3.

**Expression of PD-1 (PD-1^+^ cell proportion, PD-1 MFI) on T cells in donor PB before and after G-CSF mobilization.**

**a-b** Representative plots of the PD-1^+^ subsets in CD3^+^ and CD3^+^CD45RA^+^CCR7^+^ T cells **(a)**, CD4^+^ and CD4^+^CD45RA^+^CCR7^+^ T cells **(b)** before (D0) and after three (D3) or five days (D5) of G-CSF mobilization by FCM (*n*=24). **c-d** The percentages of the PD-1^+^ subsets in CD3^+^ and CD3^+^CD45RA^+^CCR7^+^ T cells **(c)**, CD4^+^ and CD4^+^CD45RA^+^CCR7^+^ T cells **(d)** on D0, D3 and D5 by FCM (*n*=24). **e-f** The percentages of the PD-1^+^ subsets and MFI of PD-1 in CD8^+^CD45RA^+^CCR7^-^ T cells **(e)** and CD8^+^CD45RA^-^ T cells **(f)** on D0, D3 and D5 by FCM (*n*=80). Data are presented as the mean ± SEM, **P* < 0.05, ***P* < 0.01, ****P* < 0.001, *****P* < 0.0001.


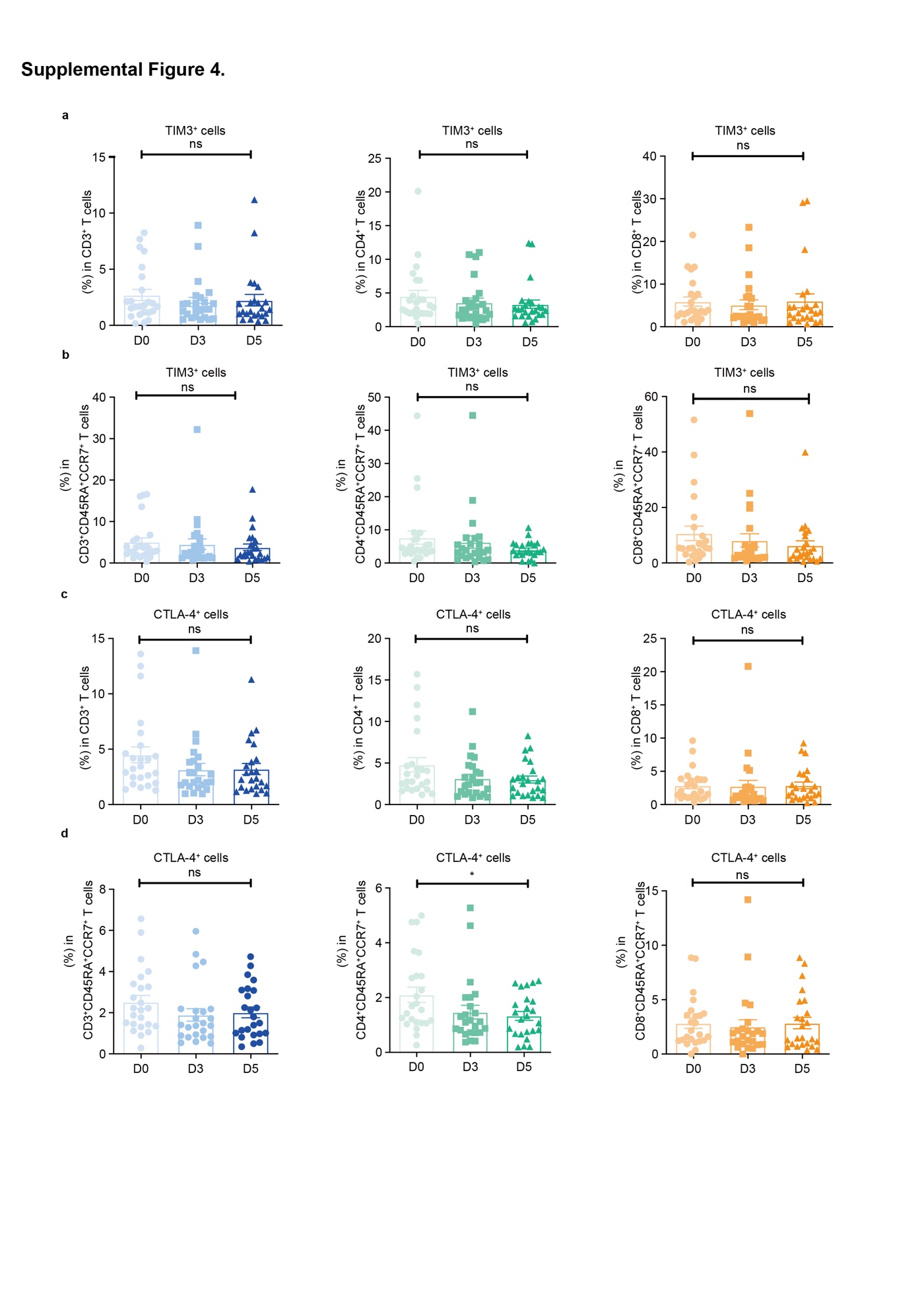


Figure. S4.

**Proportion of TIM3^+^ and CTLA-4^+^ cells in T cell subsets in donor PB before and after G-CSF mobilization.**

**a** Percentages of TIM3^+^ cells in CD3^+^ (blue), CD4^+^ (green), and CD8^+^ T (orange) cells in the PB of healthy donors before (D0) and after (D3, D5) G-CSF mobilization, respectively by FCM (*n*=24). **b** Percentages of TIM3^+^ cells in CD3^+^CD45RA^+^CCR7^+^ (blue), CD4^+^CD45RA^+^CCR7^+^ (green), and CD8^+^CD45RA^+^CCR7^+^ (orange) T cells of D0, D3 and D5, respectively by FCM (*n*=24). **c** Percentages of CTLA-4^+^ cells in CD3^+^ (blue), CD4^+^ (green), and CD8^+^ T (orange) cells of D0, D3 and D5, respectively by FCM (*n*=24). **d** Percentages of CTLA-4^+^ cells in CD3^+^CD45RA^+^CCR7^+^ (blue), CD4^+^CD45RA^+^CCR7^+^ (green), and CD8^+^CD45RA^+^CCR7^+^ (orange) T cells of D0, D3 and D5, respectively by FCM (*n*=24). Data are presented as the mean ± SEM, **P* < 0.05.


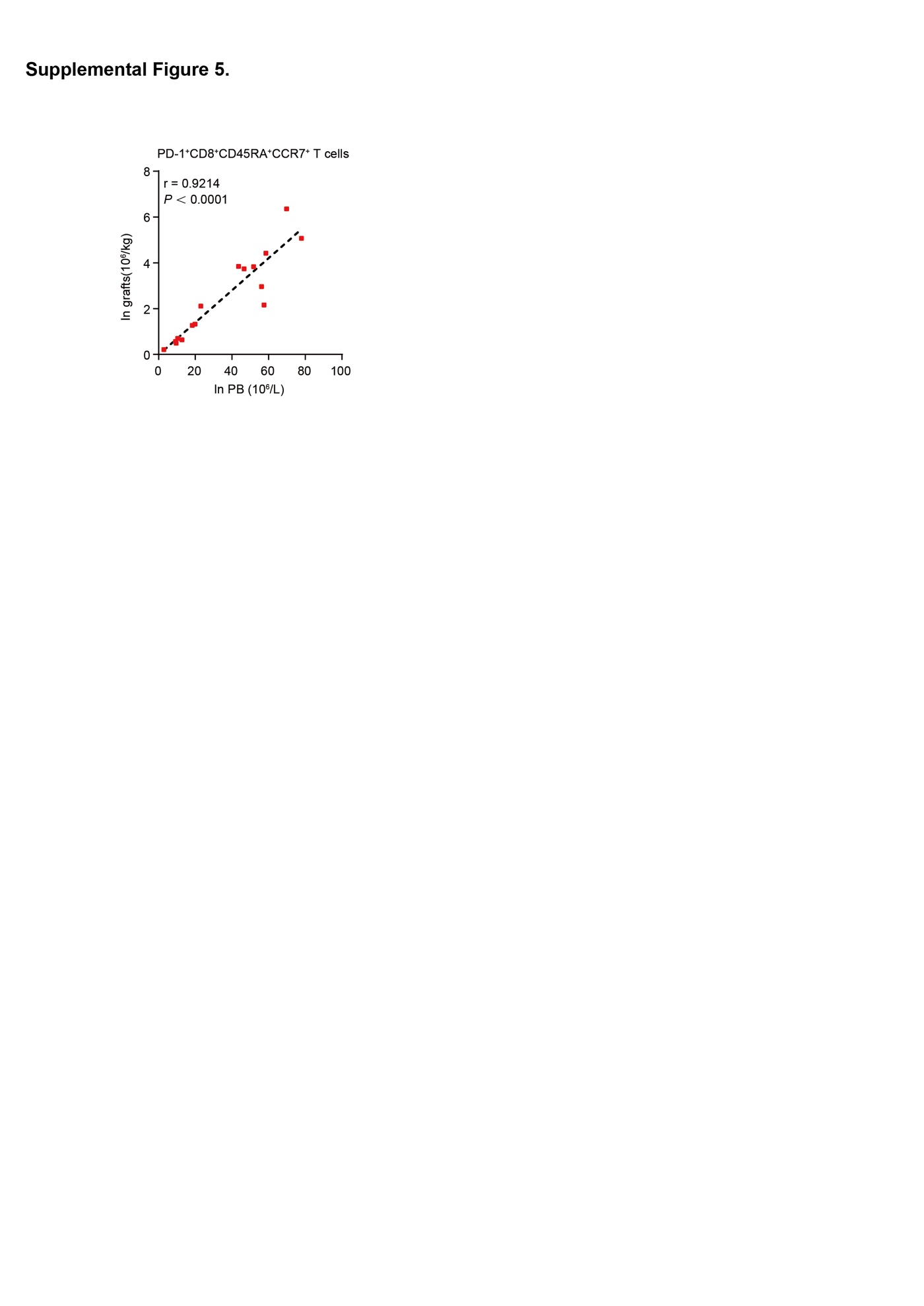


Figure. S5.

**Correlation between the PD-1^+^CD8^+^CD45RA^+^CCR7^+^ T cell counts in the grafts and those in donor peripheral blood.**


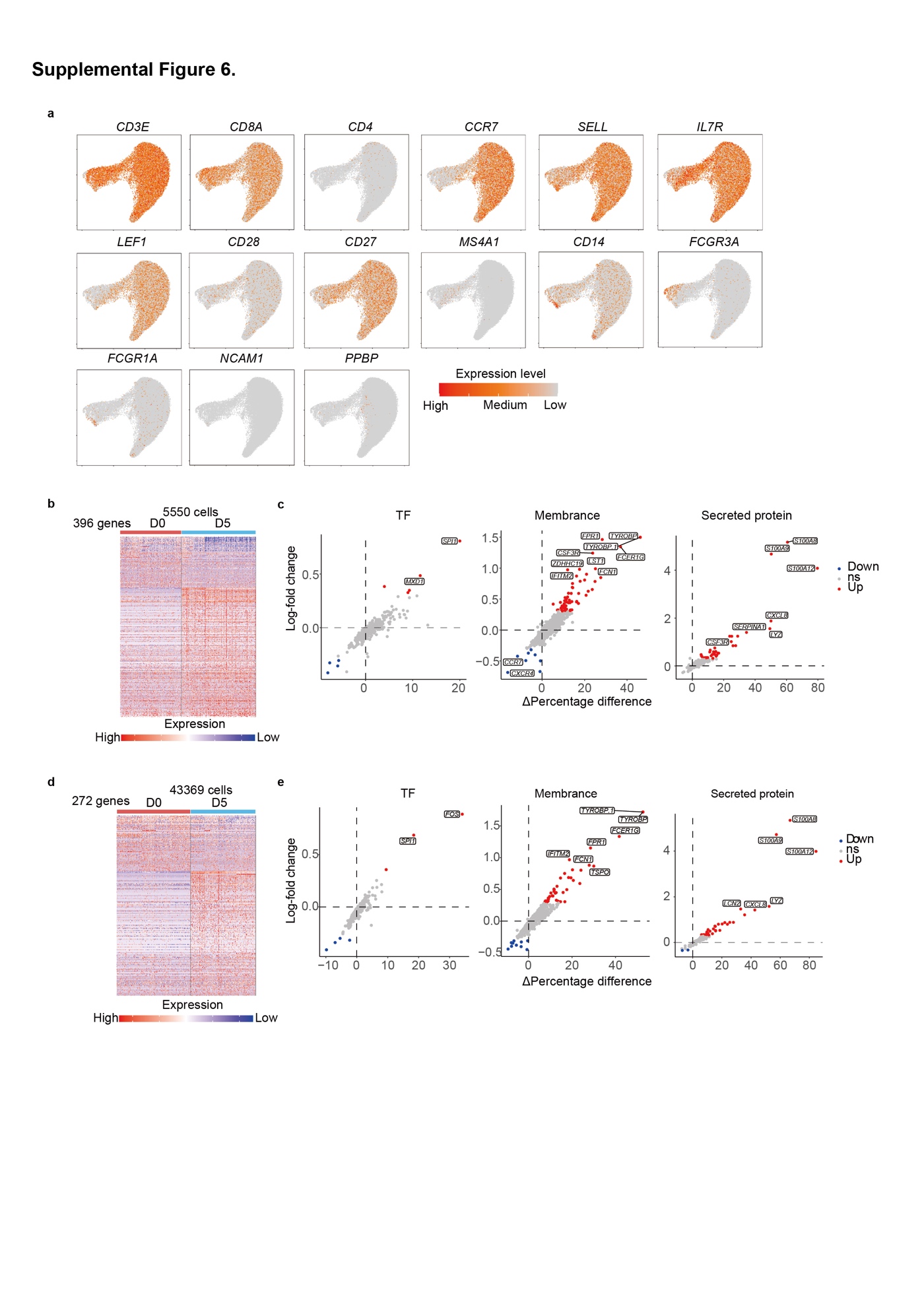


Figure. S6.

**The expression of signature genes in CD8^+^CD45RA^+^CCR7^+^ T cells, related to Figure 3.**

**a** Feature plots of key gene expressions, including CD3, CD8A, CD8B, CCR7 for CD8^+^CCR7^+^T cells, and CD4 (CD4^+^ T cell), MS4A1 (B cells), CD14, FCGR3A (monocyte), FCER1A (DC), NCAM1 (NK), PPBP (platelet), in total CD8^+^CD45RA^+^CCR7^+^ T cells. **b** Heatmap of the 396 signature genes in PD-1^+^CD8^+^CCR7^+^ cells before (D0) or after (D5) G-CSF mobilization (112 genes in the D0 group and 284 genes in the D5 group). **c** Differential gene expression analysis using the log-fold change expression versus the difference in the percentage of cells expressing the gene between PD-1^+^CD8^+^CCR7^+^ cells in D0 and D5 (Δ Percentage difference). Genes with log-fold change > 0.3, Δ Percentage difference > 10%, and adjusted *P*-value from Wilcoxon test <0.05 were labeled, including TF binding with the promotor of G-CSF receptor gene (*SPI1*) and genes associated with host defense (*FCN1, LYZ*). **d** Heatmap of the 272 signature genes in PD-1^-^CD8^+^CCR7^+^ cells in D0 and D5 (84 genes in the D0 group and 188 genes in the D5 group). **e** Differential gene expression analysis using the log-fold change expression versus the difference in the percentage of cells expressing the gene between PD-1^-^CD8^+^CCR7^+^ cells in D0 and D5 (Δ Percentage difference). Genes with log-fold change > 0.3, Δ Percentage difference > 10%, and adjusted *P*-value from Wilcoxon test < 0.05 were labeled, including TF binding with the promotor of G-CSF receptor gene (*SPI1*) and genes associated with host defense (*FCN1, LYZ*).


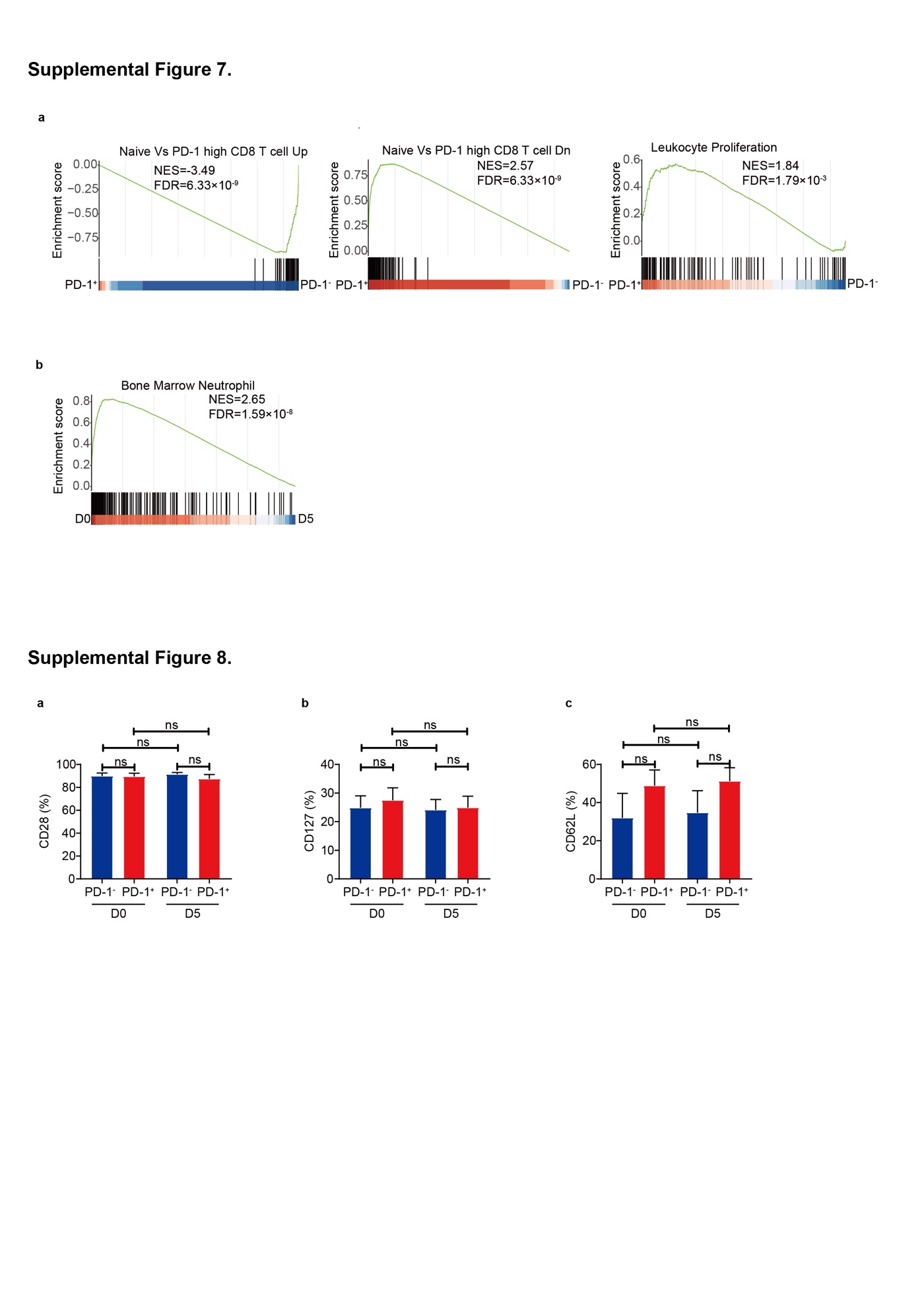


Figure. S7.

**The GSEA analysis in CD8^+^CD45RA^+^CCR7^+^ T cells, related to Figure 3.**

**a** GSEA for comparing the enrichment of differentially expressed genes between PD-1^+^CD8^+^CCR7^+^ cells and PD-1^-^CD8^+^CCR7^+^ cells in the genes up and down-regulated in comparison of CD8^+^ T_N_ versus PD-1 high CD8 T cells (accession GEO: GSE26495), and genes associated with leukocyte proliferation in GO Biological Process. **b** GSEA for comparing the enrichment of differentially expressed genes between PD-1^-^CD8^+^CCR7^+^ cells before (D0) or after (D5) G-CSF mobilization in the genes associated with cell type signature of bone marrow neutrophil.


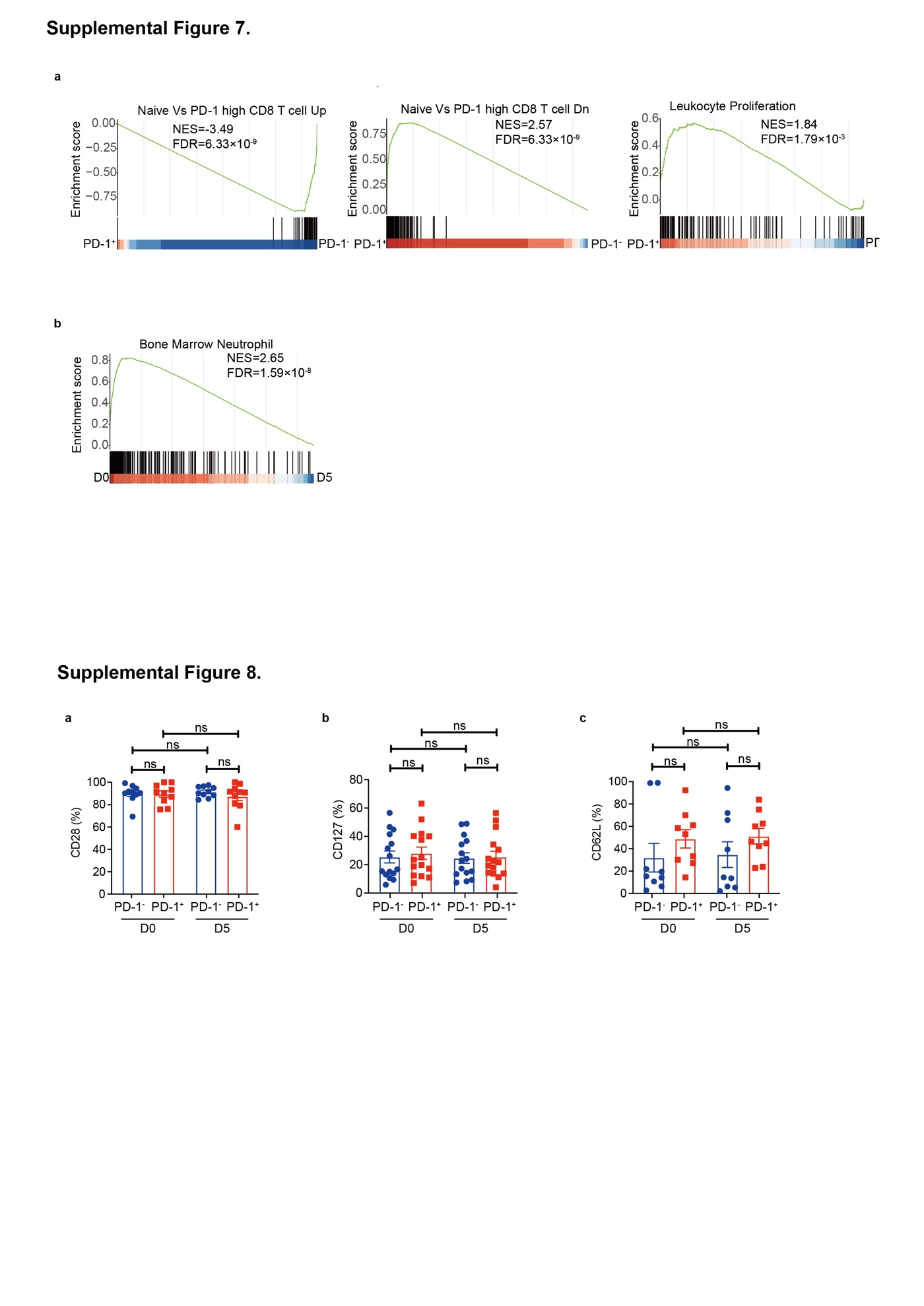


Figure. S8.

**Phenotyping the PD-1^+^ and the PD-1^-^ subsets in PB CD8^+^CD45RA^+^ CCR7^+^ T cells before and after G-CSF mobilization for activation markers.**

**a-c** Percentages of CD28^+^ **(a)**, CD127^+^ **(b)** and CD62L^+^ **(c)** cells in PD-1^-^ (blue) or PD-1^+^ (red) subsets of CD8^+^CD45RA^+^CCR7^+^ T cells before (D0) or after (D5) G-CSF mobilization (CD28: *n*=10; CD127: *n*=15; CD62L: *n*=9). Data are presented as the mean ± SEM.


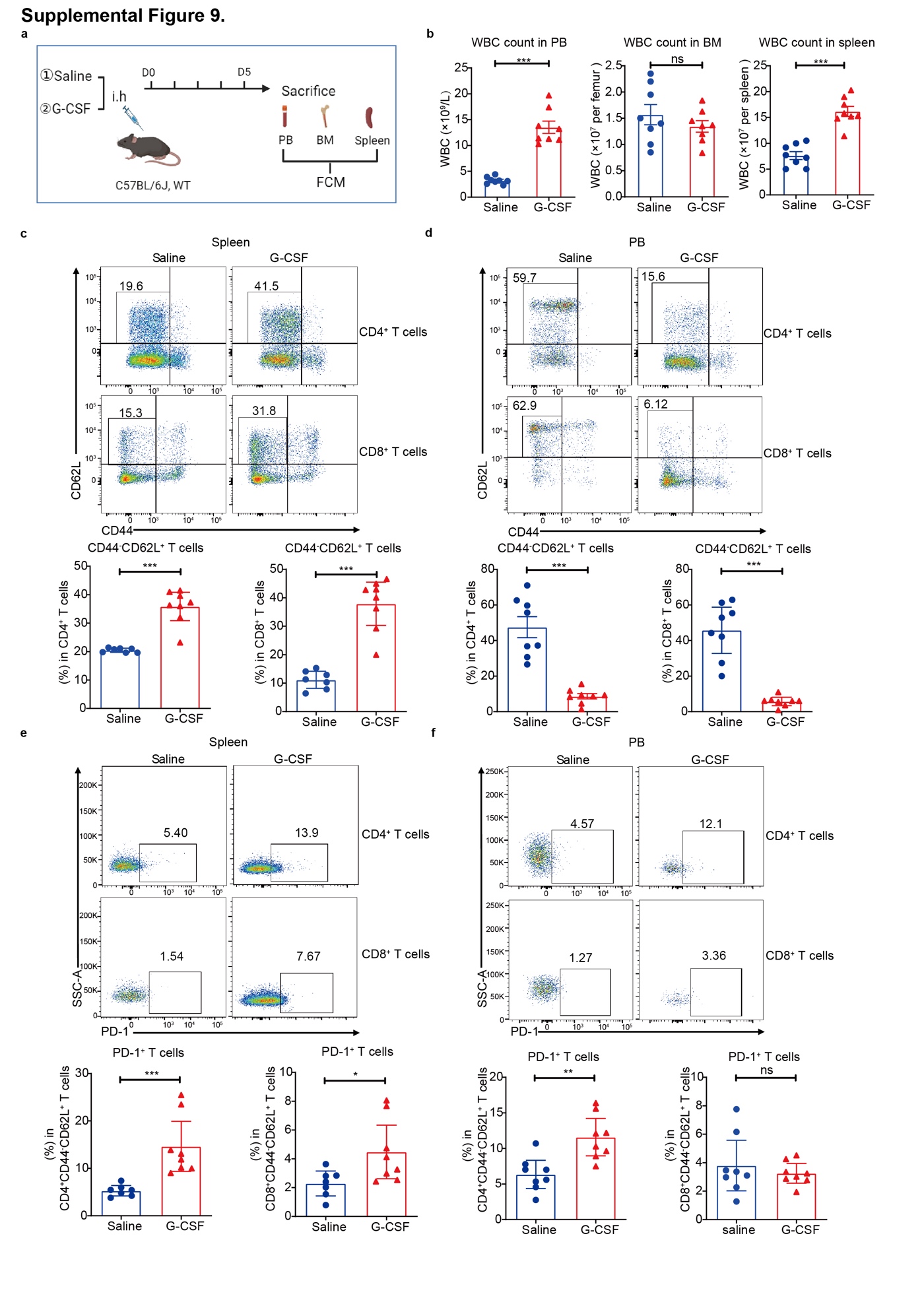


Figure. S9.

**G-CSF mobilization increases the proportion of splenic PD-1^+^CD8^+^ T_SCM_-like regulatory T cells in mice.**

**a** Outline of G-CSF mobilization in mice: rhG-CSF was subcutaneously (i.h) injected into C57BL/6J mice for 5 days, mimicking clinical G-CSF mobilization in human donors. The image was created using Biorender (<https://biorender.com/>). **b** WBC counts in the PB, spleen, and bone marrow (*n*=8) after injecting saline (blue) or rhG-CSF (red). **c** Representative plots and the percentages of CD44^-^CD62L^+^ subsets in splenic CD4^+^ and CD8^+^ T cells after injecting saline (blue) or rhG-CSF (red) by FCM (*n*=7-8)**.** **d** Representative plots and the percentages of CD44^-^CD62L^+^ subsets in PB CD4^+^ and CD8^+^ T cells after injecting saline (blue) or rhG-CSF (red) by FCM (*n*=8)**.** **e** Representative plots and the percentages of PD-1^+^ subsets in splenic CD4^+^CD44^-^CD62L^+^ and CD8^+^CD44^-^CD62L^+^ T cells after injecting saline (blue) or rh-G-CSF (red) by FCM (*n*=7-8). **f** Representative plots and the percentages of PD-1^+^ subsets in PB CD4^+^CD44^-^CD62L^+^ and CD8^+^CD44^-^CD62L^+^ T cells after injecting saline (blue) or rhG-CSF (red) by FCM (*n*=8). Data are presented as the mean ± SEM, **P* < 0.05, ***P* < 0.01, ****P* < 0.001.


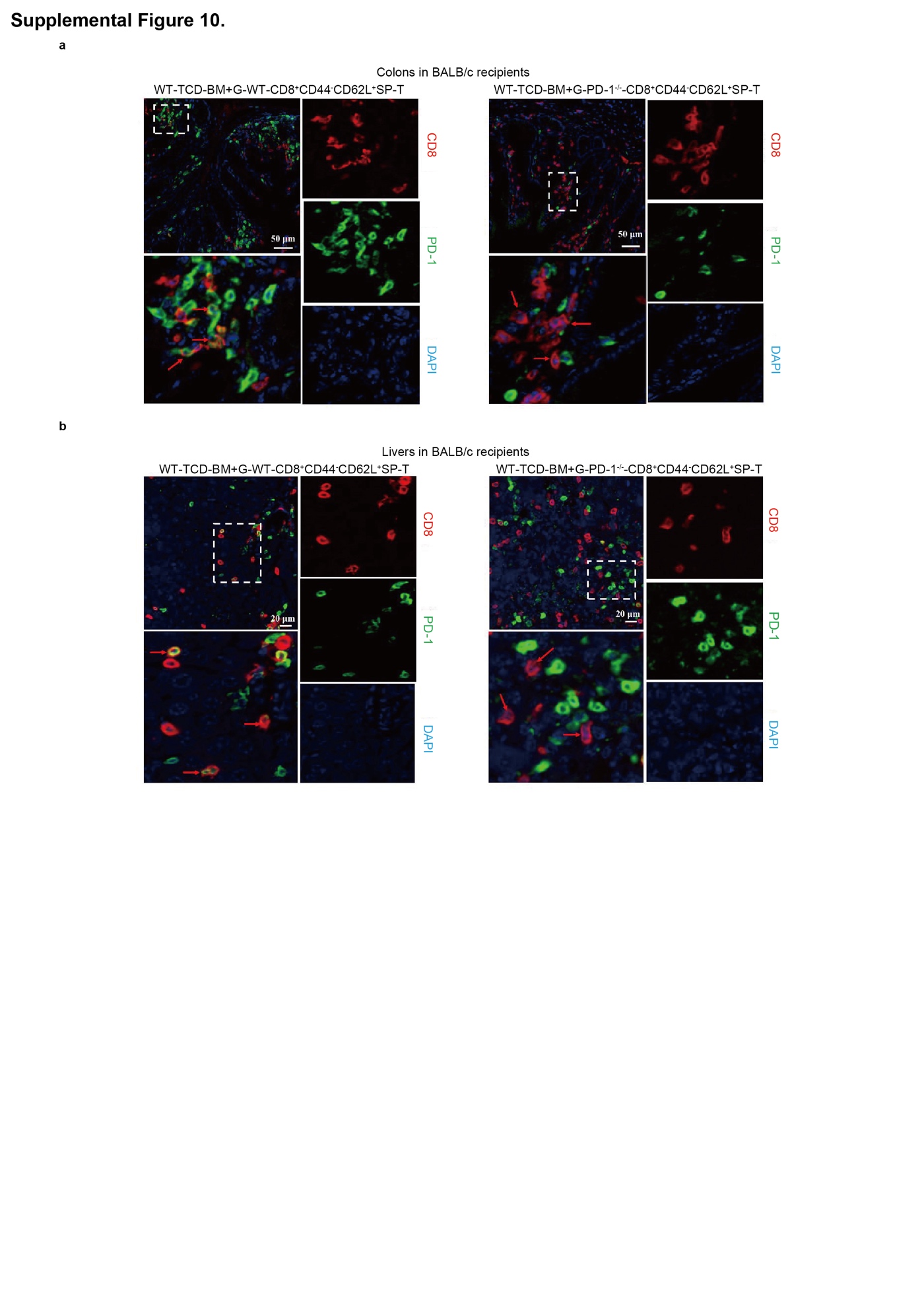


Figure. S10.

**Immunofluorescence staining of aGVHD target organs on 47 days after allogeneic HSCT.**

**a-b** Immunofluorescence staining for CD8 (red) and PD-1 (green) of colon **(a)** and liver **(b)** sections from BALB/c recipients that received T cell-depleted bone marrow (TCD-BM) cells from C57BL/6J WT donors plus G-CSF-treated splenic CD8^+^CD44^-^CD62L^+^ T cells from either C57BL/6J WT or PD-1^-/-^ mice. The tissues were collected at the onset of GVHD on day 47. Nuclei are stained by DAPI (4ʹ,6-diamidino-2-phenylindole). Scale bars, 50 μm for the colon and 20 μm for the liver.


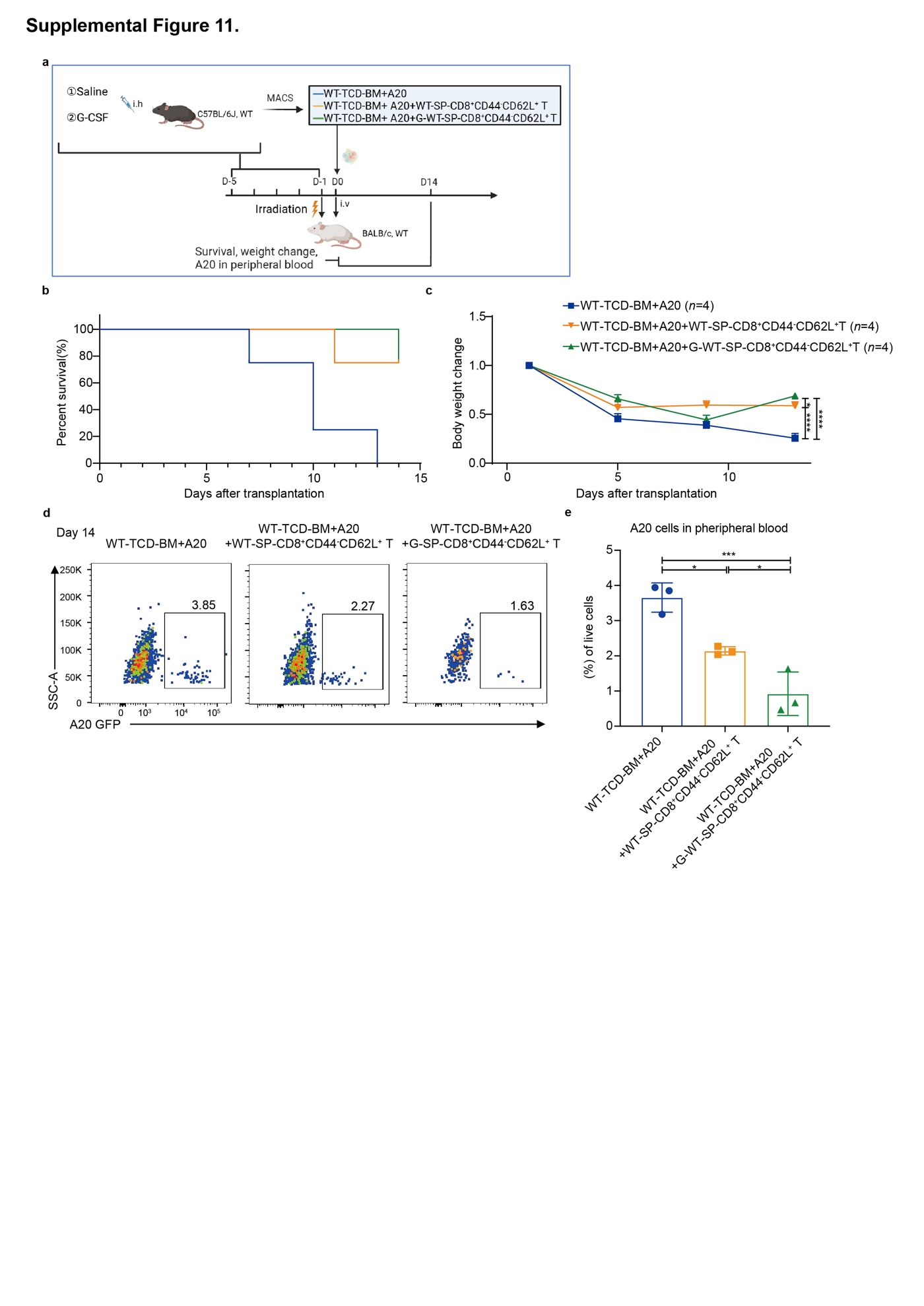


Figure. S11.

**G-CSF-induced PD-1^+^CD8^+^ T_SCM_-like regulatory cells retain GVL effect of recipient mice in the allogeneic HSCT model.**

**a** Outline shows the workflow of the GVL model. The image was created using Biorender (<https://biorender.com/>). **b-c** Body weight change and survival rate of recipient mice received WT TCD-BM along with 5×10^5^ A20 cells (blue); WT TCD-BM, 5×10^5^ A20 and WT CD8^+^CD44^-^CD62L^+^ T cells (yellow); WT TCD-BM, 5×10^5^ A20 and G-CSF-treated WT CD8^+^CD44^-^CD62L^+^ T cells (green) monitored for 14 days after transplantation (*n*=4). **d** Representative plots of A20 cell expression in peripheral blood of recipient mice on day 14 after transplantation. **e** The percentage of A20 cells in peripheral blood of recipient mice received WT TCD-BM along with 5×10^5^ A20 cells (blue); WT TCD-BM, 5×10^5^ A20 and WT CD8^+^CD44^-^CD62L^+^ T cells (yellow); WT TCD-BM, 5×10^5^ A20 and G-CSF-treated WT CD8^+^CD44^-^CD62L^+^ T cells (green) on day 14 after transplantation by FCM (*n*=3). Data are presented as the mean ± SD, **P* < 0.05, ****P* < 0.001, *****P* < 0.0001.


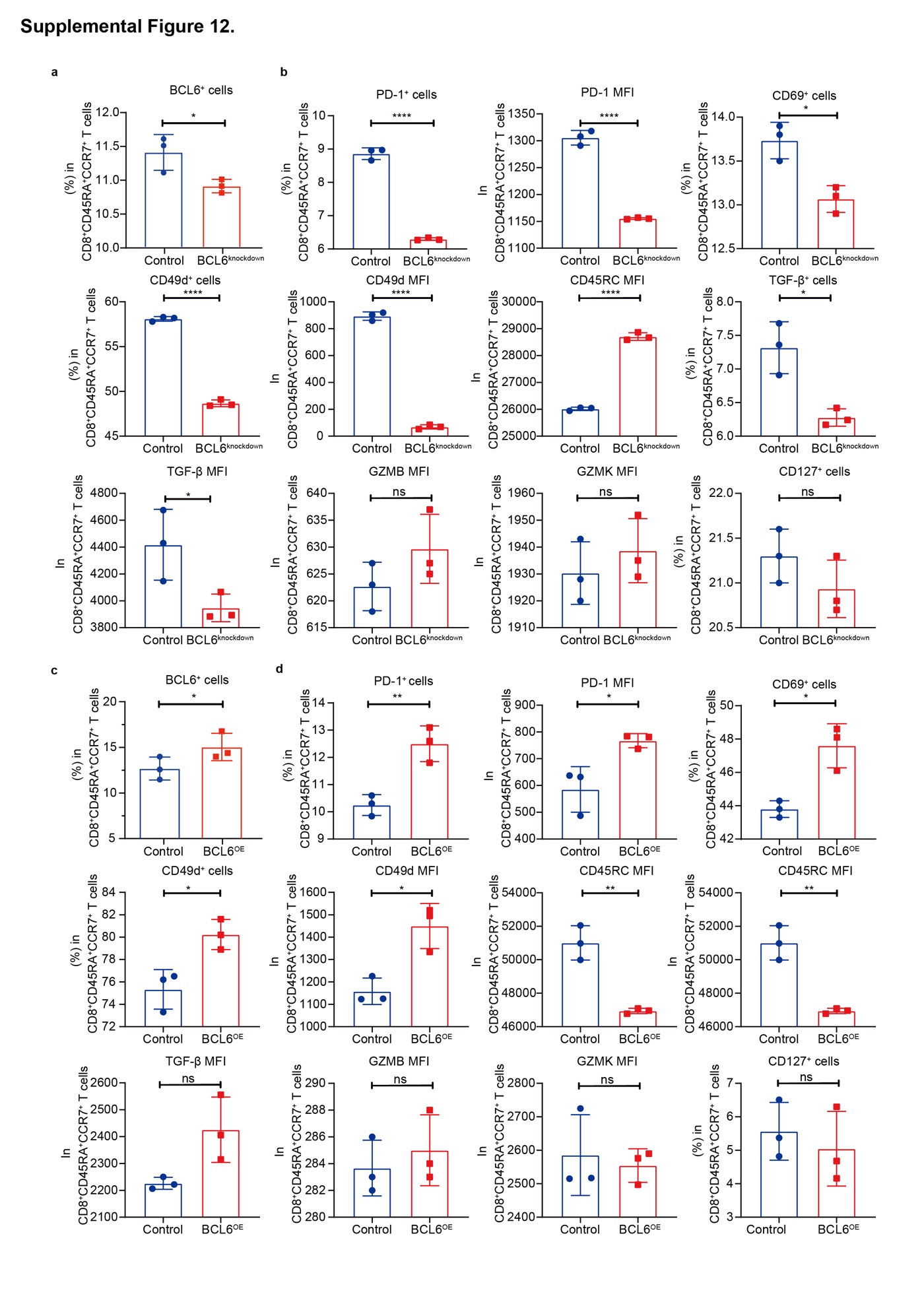


Figure. S12.

**Phenotypes of BCL6-knockdown CD8^+^CD45RA^+^CCR7^+^ T cells and BCL6-overexpression CD8^+^CD45RA^+^CCR7^+^T cells from healthy human PB analyzed by FCM.**

**a** BCL6 expression in control (blue) and BCL6-knockdown (red) CD8^+^CD45RA^+^CCR7^+^ T cells from healthy human PB by FCM (*n*=3). **b** PD-1, CD69, CD49D, CD45RC, TGF-β, GZMB, GZMK and CD127 expression (percentage and MFI) in control (blue) and BCL6-knockdown (red) CD8^+^CD45RA^+^CCR7^+^ T cells from healthy human PB by FCM (*n*=3). **c** BCL6 expression in control (blue) and BCL6-overexpression (red) CD8^+^CD45RA^+^CCR7^+^ T cells from healthy human PB by FCM (*n*=3). **d** PD-1, CD69, CD49D, CD45RC, TGF-β, GZMB, GZMK and CD127 expression (percentage and MFI) in control (blue) and BCL6-overexpression (red) CD8^+^CD45RA^+^CCR7^+^ T cells from healthy human PB by FCM (*n*=3). Data are presented as the mean ± SD, **P* < 0.05, ***P* < 0.01, *****P* < 0.0001.


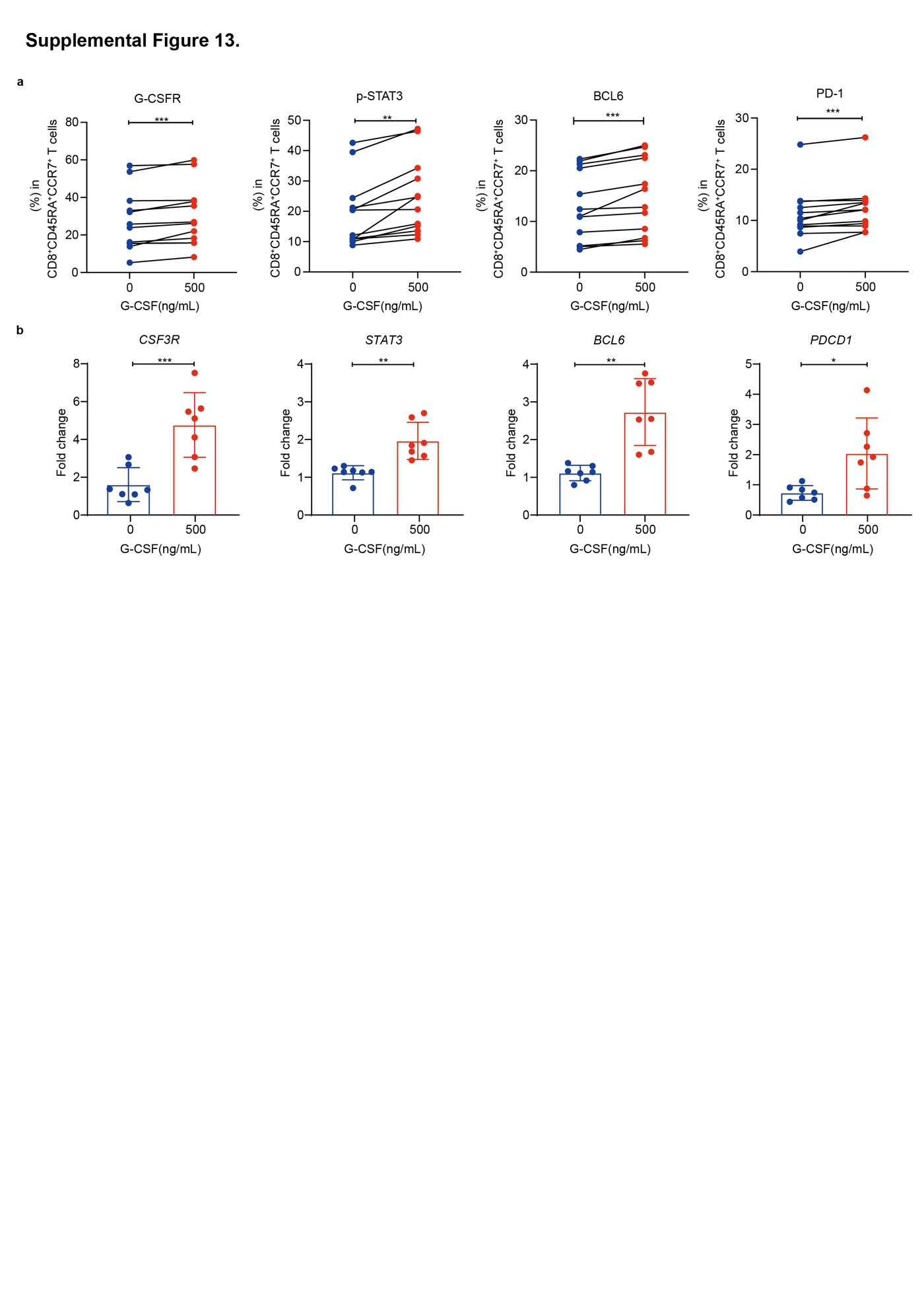


Figure. S13.

**G-CSF treatment** **increased G-CSFR, STAT3, BCL6, and PD-1 expression (protein and mRNA levels) in CD8^+^CD45RA^+^CCR7^+^ T cells from healthy human PB *in vitro* analyzed by FCM and q-PCR.**

**a** G-CSFR, p-STAT3, BCL6, and PD-1 protein expression in CD8^+^CD45RA^+^CCR7^+^ T cells from healthy human PB after stimulation with (red) or without (blue) 500 ng/ml G-CSF *in vitro* for 48 hours by FCM (*n*=12). **b** *CSFR3*, *STAT3*, *BCL6,* and *PDCD1* mRNA expression in CD8^+^CD45RA^+^CCR7^+^ T cells from healthy human PB after stimulation with (red) or without (blue) 500 ng/ml G-CSF *in vitro* for 48 hours by q-PCR (*n*=7). Data are presented as the mean ± SD, **P* < 0.05, ***P* < 0.01, ****P* < 0.001.

Table S1.

| **Supplemental Table 1. Information of donors and recipients in allogeneic HSCT** | |
| --- | --- |
| **Characteristics** | **All recipients (n = 80)** |
| **Number** | 80 |
| **Donor gender (male/female, %)** | 60 (75%) / 20(25%) |
| **Donor age (years, median, range)** | 36 (10-57) |
| **Recipient gender (male/female, %)** | 47 (58.75%) / 33(41.25%) |
| **Recipient age (years, median, range)** | 37 (5-63) |
| **Pretransplant diagnosis** |  |
| AML | 36 (45%) |
| ALL | 10(12.5%) |
| CML | 2 (2.5%) |
| CLL | 1 (1.25%) |
| MDS | 11 (13.75%) |
| Lymphoma | 5 (6.25%) |
| Other hematologic diseases | 15 (18.75%) |
| **Donor match** |  |
| HLA-identical donor | 22 (27.5%) |
| HLA-haploidentical donor | 58 (72.5%) |
| **Stem cell source** |  |
| PBSCs | 80 (100%) |
| BM and PBSCs | 0 (0.0%) |
| **Donor/recipient sex match** |  |
| Male-to-male | 34 (42.5%) |
| Male-to-female | 26 (32.5%) |
| Female-to-male | 12 (15%) |
| Female-to-female | 8 (10%) |
| **Donor–recipient ABO blood type** |  |
| Match | 52 (65%) |
| Minor mismatch | 16 (20%) |
| Major mismatch | 12 (15%) |
| **Donor–recipient relation** |  |
| Parent-to-child | 21 (26.25%) |
| Sibling-to-sibling | 34 (42.5%) |
| Child-to-parent | 25 (31.25%) |
| **Cells in allograft** |  |
| Monocytes (×10^8^/kg), median (range) | 6.86 (2.29-8.2) |
| CD34^+^ cells (×10^6^/kg), median (range) | 9.37 (3.68-9.72) |
| CD3^+^ cells (×10^8^/kg), median (range) | 5.335 (1.60-6.13) |
| **Grade of GVHD** |  |
| Grade 1 | 5 (33.33%) |
| Grade 2-4 | 10 (66.67%) |
| **GVHD organ involvement** |  |
| Skin | 11 (73.33%) |
| Liver | 4 (26.67%) |
| GI | 5 (33.33%) |
| **Median days to aGVHD (range)** | 20 (13 -70) |

**Abbreviations:** AML, acute myeloid leukemia; ALL, acute lymphoid leukemia; CML, chronic myeloid leukemia; CLL, Chronic lymphoblastic leukemia; MDS, Myelodysplastic syndrome; HLA, human leukocyte antigen; GVHD, graft-versus-host disease; GI, Gastrointestinal.

Table S2.

| **Supplementary Table 2. Impact of PD-1^+^CD8^+^CD45RA^+^CCR7^+^ T cells on post-transplant outcomes** | | | | | |
| --- | --- | --- | --- | --- | --- |
| **Variable** | **Outcomes** | **Low PD-1^+^ group** | **High PD-1^+^ group** | | ***P* value** |
| **ALL** | aGVHD | 23.81±6.148 | 3.704±1.852 | **0.0096** | |
|  | Graft failure | 6.944±2.6 | 8.226±1.674 | 0.8084 | |
|  | Infection | 37.04±18.52 | 16.13±8.065 | **0.0120** | |
|  | cGVHD | 6.694±2.66 | 4.608±1.19 | 0.7067 | |
|  | Relapse | 10±3.239 | 8.737±1.618 | 0.9138 | |
|  | Death | 15.87±4.098 | 10.37±1.755 | 0.5408 | |
| **HLA-haploidentical transplantation** | aGVHD | 23.81±6.148 | 7.337±1.740 | **0.0398** | |
|  | Graft failure | 10.42±3.989 | 7.764±1.948 | 0.7228 | |
|  | Infection | 33.33±16.67 | 17.39±8.696 | 0.1139 | |
|  | cGVHD | 5.556±2.778 | 5.072±1.449 | ＞0.9999 | |
|  | Relapse | 15±4.859 | 9.565±1.977 | 0.5116 | |
|  | Death | 15±4.859 | 12.88±2.276 | 0.9297 | |
| **HLA-identical transplantation** | aGVHD | 30±9.718 | 4.167±2.083 | **0.0080** | |
|  | Graft failure | 0 | 11.25±3.644 | 0.2733 | |
|  | Infection | 44.44±22.22 | 12.50±6.25 | **0.0358** | |
|  | cGVHD | 11.11±5.556 | 4.167±2.083 | 0.5093 | |
|  | Relapse | 0 | 7.813±2.992 | 0.3789 | |
|  | Death | 20.83±7.979 | 4.167±2.083 | 0.1374 | |

Table S3.

| **Supplementary Table 3. Performance of PD-1% results stratified by various variables** | | | | |
| --- | --- | --- | --- | --- |
| **Variable** | **PD-1^+^CD8^+^CD45RA^+^CCR7^+^ %>3.4 (%)** | **Sensitivity%**  **(95%CI)** | **Specificity%**  **(95%CI)** | **AUC** |
| **All** |  |  |  |  |
| Male, ≤ 36 | 81.5 | 66.7 | 87.5 | 0.67 |
| Male, > 36 | 75.8 | 50 | 84 | 0.63 |
| Female, ≤ 36 | 61.5 | 50 | 66.7 | 0.61 |
| Female, > 36 | 100 | NA | 100 | NA |
| **HLA-haploidentical donor** |  |  |  |  |
| Male, ≤ 36 | 81 | 66.7 | 88.9 | 0.69 |
| Male, > 36 | 80.8 | 40 | 85.7 | 0.62 |
| Female, ≤ 36 | 66.7 | 33.3 | 66.7 | 0.5 |
| Female, > 36 | 100 | NA | 100 | NA |
| **HLA-identical donor** |  |  |  |  |
| Male, ≤ 36 | 83.3 | NA | 83.3 | NA |
| Male, > 36 | 57.1 | 66.7 | 75 | 0.5 |
| Female, ≤ 36 | 50 | 100 | 66.7 | 0.67 |
| Female, > 36 | 100 | NA | 100 | NA |

**Abbreviations:** AUC, Area under curve; NA, not available.

Table S4.

| **Supplemental Table 4. Antibodies for FCM and primers for q-PCR** | | | |
| --- | --- | --- | --- |
| **Antibodies for FCM** | | | |
| **Reactivity** | | **Markers** | **Fluorescence** |
| Human | | CD45 | AF700 |
|  |  | CD3 | APC/APC-Cy7 |
|  |  | CD4 | BUV496/APC-Cy7 |
|  |  | CD8 | BUV395/PE |
|  |  | CD45RA | BV510/AF700 |
|  |  | CCR7 | APC/PE-CF594 |
|  |  | PD-1 | PE-Cy7/APC |
|  |  | TIM3 | BV421 |
|  |  | CTLA-4 | PE |
|  |  | CD103 | APC-Cy7 |
|  |  | CD39 | Percp-Cy5.5 |
|  |  | CD73 | APC-Cy7 |
|  |  | CXCR3 | FITC |
|  |  | CD95 | PE-Cy7 |
|  |  | CD45RC | APC |
|  |  | CD69 | FITC |
|  |  | CD49d | PE |
|  |  | CD28 | PE-CF594 |
|  |  | CD127 | FITC |
|  |  | CD62L | BV605 |
|  |  | IL-10 | BV421 |
|  |  | TGF-β | FITC |
|  |  | CD107a | BV650 |
|  |  | Perforin | BV711 |
|  |  | Granzyme B | PE-Cy7 |
|  |  | IFN-γ | FITC |
|  |  | IL-2 | BV605 |
|  |  | BCL6 | PE-Cy7 |
|  |  | G-CSFR | PE |
|  |  | p-STAT3 | APC |
| Mouse | | CD3 | AF700 |
|  |  | CD4 | PE-Cy7/APC |
|  |  | CD8 | Percp-Cy5.5/ APC-Cy7 |
|  |  | CD44 | BV510 |
|  |  | CD62L | PE |
|  |  | PD-1 | BV421 |
| Human and mouse | | Fixable Viability Dye | BV510 |
| **Primers for q-PCR** | | | |
| **Gene** | **Sequence** | | |
| Human *CSF3R* | Forward 5’-CTTGTGGCCTATAACTCAGCC-3’ | | |
|  | Reverse 5’-CCCACTCAATCACATAGCCCT-3’ | | |
| Human *STAT3* | Forward 5’-CAGCAGCTTGACACACGGTA-3’ | | |
|  | Reverse 5’-AAACACCAAAGTGGCATGTGA-3’ | | |
| Human *BCL6* | Forward 5’-ACACATCTCGGCTCAATTTGC-3’ | | |
|  | Reverse 5’-AGTGTCCACAACATGCTCCAT-3’ | | |
| Human *PDCD1* | Forward 5’-CCAGGATGGTCTTAGACTCCCC-3’ | | |
|  | Reverse 5’-TTTAGCACGAAGCTCTCCGAT-3’ | | |
| Human *BETA-ACTIN* | Forward 5’-CATGTACGTTGCTATCCAGGC-3’ | | |
|  | Reverse 5’-CTCCTTAATGTCACGCACGAT-3’ | | |
| Human *PDCD1* promoter | Forward 5’- CCCTGATTGCCAGCTCAACT-3’ | | |
|  | Reverse 5’- AGGAAGAGGAGACTGCTACTGA-3’ | | |
| Mouse *Bcl6* | Forward 5’-GGACAGTGCTGACCCCTAAC-3’ | | |
|  | Reverse 5’-GCCACAGAAAATGCACATGGATA-3’ | | |
| Mouse *Beta-actin* | Forward 5’-GTGACGTTGACATCCGTAAAGA-3’ | | |
|  | Reverse 5’-GCCGGACTCATCGTACTCC-3’ | | |

Data S1-9. (separate file)

1. GO pathway enriched in PD-1^-^CD8^+^CCR7^+^ cells compared with PD-1^+^CD8^+^CCR7^+^ cells.csv

2. GO pathway enriched in PD-1^+^CD8^+^CCR7^+^ cells compared with PD-1^-^CD8^+^CCR7^+^ cells.csv

3. GO pathway enriched in PD-1^-^CD8^+^CCR7^+^ cells of D0 compared with PD-1^‑^CD8^+^CCR7^+^ cells of D5.csv

4. GO pathway enriched in PD-1^+^CD8^+^CCR7^+^ cells of D0 compared with PD-1^+^CD8^+^CCR7^+^ cells of D5.csv

5. GO pathway enriched in PD-1^-^CD8^+^CCR7^+^ cells of D5 compared with PD-1^-^CD8^+^CCR7^+^ cells of D0.csv

6. GO pathway enriched in PD-1^+^CD8^+^CCR7^+^ cells of D5 compared with PD-1^+^CD8^+^CCR7^+^ cells of D0.csv

7. GSEA results enriched in PD-1^-^CD8^+^CCR7^+^ cells of D5 compared with PD-1^-^CD8^+^CCR7^+^ cells of D0.csv

8. GSEA results enriched in PD-1^+^CD8^+^CCR7^+^ cells of D5 compared with PD-1^+^CD8^+^CCR7^+^ cells of D0.csv

9. GSEA results enriched in PD-1^+^CD8^+^CCR7^+^ cells compared with PD-1^‑^CD8^+^CCR7^+^ cells.csv
